# Geopolymers vs. Cement Matrix Materials: How Nanofiller Can Help a Sustainability Approach for Smart Construction Applications—A Review

**DOI:** 10.3390/nano11082007

**Published:** 2021-08-05

**Authors:** Marco Valente, Matteo Sambucci, Abbas Sibai

**Affiliations:** 1 Department of Chemical Engineering, Materials, Environment, Sapienza University of Rome, 00184 Rome, Italy; matteo.sambucci@uniroma1.it (M.S.); abbas.sibai@uniroma1.it (A.S.); 2 INSTM Reference Laboratory for Engineering of Surface Treatments, Department of Chemical Engineering, Materials, Environment, Sapienza University of Rome, 00184 Rome, Italy

**Keywords:** CO_2_ emissions, energy efficiency, Portland replacement, geopolymer concrete mixes, mechanical properties, microstructure, geopolymer nanocomposites, durability, high-temperature behavior, LCA analysis

## Abstract

In the direction of reducing greenhouse emissions and energy consumption related to the activities of the cement and concrete industry, the increasingly popular concept of eco-sustainability is leading to the development and optimization of new technologies and low impact construction materials. In this respect, geopolymers are spreading more and more in the cementitious materials field, exhibiting technological properties that are highly competitive to conventional Portland concrete mixes. In this paper, the mix design, mechanical properties, microstructural features, and mineralogical properties of geopolymer mixes are discussed, investigating the influence of the main synthesis parameters (curing regime, type of precursors, activator molarity, mix design) on the performance of the final product. Moreover, recent developments of geopolymer technology based on the integration of functional nanofillers are reported. The novelty of the manuscript is to provide a detailed collection of past and recent comparative studies between geopolymers and ordinary Portland concrete mixes in terms of strength properties, durability, fire resistance, and environmental impact by LCA analysis, intending to evaluate the advantages and limitations of this technology and direct research towards a targeted optimization of the material.

## 1. Introduction

After water, concrete is the world’s second most important substance needed for humans, but its hidden effects on the environment and health make it one of the most polluting materials on the planet. Carbon dioxide (CO_2_) emissions straightforwardly correspond to the cement content used in the concrete mix. Portland cement (PC) is the most common type of cementitious binder in general use around the world as a basic ingredient of concrete or mortars. Typical raw materials used to fabricate PC include limestone, marl, clay, slag, silica sand, and iron ores [1]. The manufacturing process of PC entails the following major steps [1,2]:

- *Raw materials preparation*. After quarrying, principal raw materials (such as limestone and clay) are crushed to a maximum of about 7.5 cm in diameter by a two-step grinding treatment. In a “dry” production method, crushed aggregates are fed directly into the kiln. Conversely, the “wet” method consists of the mixing of ground material with water to form a slurry, a suspension of creamy consistency composed of water (35–50%) and fine particles. Next, the slurry is inserted into the kiln. “Wet” preparation ensures an easier control of the chemistry process and is suitable in the presence of moist raw aggregates. However, it has higher energy requirements due to the need for slurry water evaporation. On the other hand, the “dry” process is faster and less energy expensive [3].
- *Clinker production*. Ground ingredients are burned in the kiln at a temperature of about 1350 °C to 1500 °C. Various materials can be used as kiln fuels. Traditional sub-stances are fossil fuels, oil, coal, and gas. Secondary raw materials, including waste oils, plastics, waste tires, and sewage sludge, are considered alternative fuels for the cement industry [4]. Thermal treatment involves the partial fusion of raw materials, the breaking of their chemical bonds, and the recombination into new compounds. The result is a nodular-shaped clinker product.
- *Final grinding process*. After cooling, clinker nodules are crushed into a superfine powder by steel ball milling treatment. During this process, the clinker is mixed with a small amount of gypsum (3–5%) to produce PC. Gypsum prevents the flash setting of the cement and regulates its hardening.

Specifically, PC results from the calcination of limestone (calcium carbonate or CaCO_3_) and silico-aluminous materials in burnt lime (CaO)-based products, according to the following reaction [5]:

5CaCO_3_ + 2SiO_2_ →(3CaO, SiO_2_) + (2CaO, SiO_2_) + 5CO_2_

Production of 1 ton of PC is estimated to release an average of 0.86 ton of CO_2_. Calcination is the major way of CO_2_ emission. Approximately 540 kg of CO_2_ per ton of clinker is released from this process. The rest of greenhouse emission mainly results from fuel combustion to provide the thermal energy for maintaining the high temperatures into the kiln. However, there are other secondary sources of polluting emission: CO_2_ emission from electricity consumption (from 1% to 10% of global CO_2_ releases, depending on the local energy efficiency source) and contribution of mining and transportation of the mineral raw materials (~5%) [6]. As highlighted in the De Lena et al. analysis [7], cement manufacturing is responsible for about 8% of overall anthropogenic CO_2_ emissions. This chemical compound is the most abundant component among all greenhouse gasses, having the highest impact on the global warming phenomenon. Benhelal et al. [8] report that the increase in the global mean temperature could led to catastrophic environmental consequences, such as extinction of many animal and plant species, risks for biodiversity hotspots and ecosystems, extremely adverse weather events, and damage associated with excessive sea level rise.

In this framework, how to minimize the dangerous effect of the cement industry in terms of atmospheric pollution and energy saving, has become an urgent question for re-searchers. Several low-carbon strategies were proposed [9]:

- Use of alternative fuels or raw materials to mitigate CO_2_ emission for Portland cement manufacturing.
- Adoption of CO_2_-capturing technologies in the cement plants.
- Development of Portland-free alternative binders.

This last category includes geopolymers, a new class of ceramic-cementitious mixes. Geopolymers have gained strong interest in the construction materials sector as an alternative to ordinary cement thanks to its sustainability characteristics and promising engineering performance. After a brief overview of the low-emission strategies currently applied in the cement and concrete industry (Section 2), geopolymer technology will be investigated by analyzing the properties and performance as a function of the parameters involved in their production (Section 3). According to the journal’s proposal, with the aim of exploring the recent advances of geopolymer technology, an in-depth study was dedicated to the optimization of the mixes with nanostructured fillers. In Section 4, to investigate the functionality of the geopolymer-based materials, a collection of comparative studies with conventional Portland mixes is reported in terms of mechanical properties, durability properties, resistance to high temperatures, and environmental impact.

## 2. CO_2_ Emission Mitigation Strategies in the Cement Industry: An Overview

### 2.1. Alternative Fuels in PC Manufacturing

**Figure 1:**
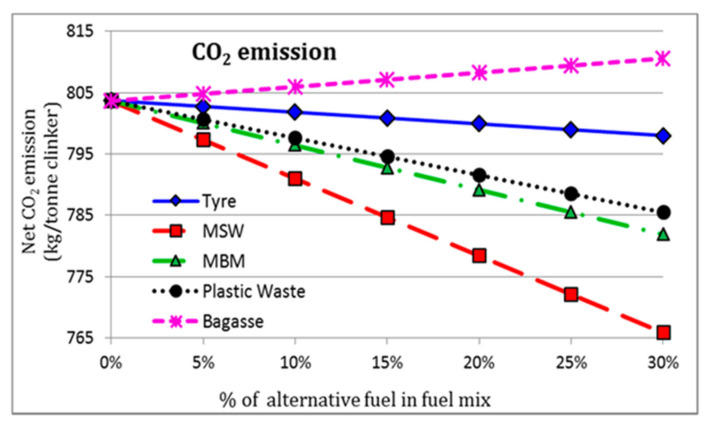
Net CO_2_ emission for a different feed rate of waste-deriving alternative fuels: used tyres, municipal solid waste (MSW), meat and bone meal (MBM), plastic waste, and bagasse [12].

Adoption of biomass or industrial wastes (tires, sludge, waste oil, plastics, fabrics, etc.) in cement manufacturing, as a replacement of conventional fuels, is in progress in most countries [9,10]. These materials, with a high Carbon-to-Hydrogen (C-H) ratio, can remarkably reduce CO_2_ emission compared to conventional fossil fuels. Other benefits are related to the preservation of non-renewable sources, reduction of landfill sites, and maximization of energy recovery from wastage [9,11]. To demonstrate the eco-efficiency performance of alternative fuels in the cement industry, Rahman et al. [12] proposed a numerical comparative analysis on the employment of some types of waste products (specifically tire, plastics, wastes of animal origin, and municipal solid wastes) as substitutes for traditional fossil fuels in the PC production. The net CO_2_ release (kg/ton of clinker) is illustrated in Figure 1 for different feed content of alternative material in the fuel mix, where 0% of alternative fuel indicates the carbon emission in the case of traditional coal fuel.

The overall decrease in CO_2_ emissions justifies the high efficiency of waste-deriving alternative fuels in terms of low environmental impact. In fact, the results indicated that up to a 4.4% reduction in CO_2_ emissions and up to a 6.4% reduction in thermal energy requirement could be achieved using these alternative fuels with 20% mix in coal without significantly altering the clinker quality [12]. However, careful controls are required for the integration of these resources during the clinkering process. Inhomogeneous thermal distribution in the kiln, higher emissions of harmful compounds/elements (Carbon monoxide, Sulphur dioxide, Nitrogen oxides, Chloride, heavy metals), and possible accumulation of dust in the apparatus are some of the principal limitations of this approach. Besides, switching from conventional fuel to alternative ones requires extensive economic investments related to the technical modification of the plant and the implementation of new fuels storage and distribution systems [10]. 

### 2.2. CO_2-_Capturing Technologies

Generally, capture and storage technologies (CSTs) refer to a set of methodologies designed to separate the CO_2_ produced by combustion and then to compress, transport, and inject it into geological formations. In highly efficient cement plants where the fuel consumption is significantly reduced and the potential for improving energy performance is very limited, the integration of CSTs can produce a drastic reduction of CO_2_ emission coming from the calcination reaction [13]. Below, a brief description of the main post-combustion CO_2_ capture systems is proposed. Pre-combustion solutions, mainly intended for the mitigation of fuel-derived CO_2_ [3], are not addressed in this paper.

#### 2.2.1. Post-Combustion Amine Scrubbing

This method refers to CO_2_ removal from the calcination flue gas by using an amine-based chemical solvent such as Monoethanolamine (MEA). Post-combustion gaseous products pass through the aqueous amine solvent, which binds chemically with the CO_2_. The solvent loaded with CO_2_ (designated as “rich” solvent) is heated up above 120 °C in a regenerator reactor where the reverse CO_2_-amine reaction occurs. The process releases pure CO_2_ and regenerates the amine solvent. Such compound (“lean” solvent) is recycled back to restart the capturing process, while the pure CO_2_ is compressed to an adequate pressure (110 bar) for efficient transportation and storage. A CO_2_ reduction efficiency of around 90% is estimated by using amine-based capturing technology [14]. However, several limitations must be considered [3,14]:

- Adverse effect of the gaseous and solid clinkering by-products including Nitrogen dioxide (NO_2_), Sulphur dioxide (SO_2_), Hydrochloric acid (HCl), and dust on the absorption efficiency of amine solvent.
- Additional amount of heat required to regenerate the solvent. This contribution remarkably penalizes the power plant efficiency.
- The large-scale application of amine-based systems is opposed by the toxicity of the amine chemical products.

#### 2.2.2. Calcium (Ca) Looping Process 

In this capture technology, a regenerable limestone-derived CaO sorbent is used to separate CO_2_ from cement kiln flue gas through sequential carbonation-calcination cycles. The process is performed in two interconnected reactors operating at atmospheric pressure: carbonator and calciner. In the carbonator, CaO sorbent removes CO_2_ from the gaseous phase at high thermal conditions (600–750 °C). According to exothermic carbonation reaction, solid Calcium Carbonate (CaCO_3_) is formed:

CaO + CO_2_ → CaCO_3_

In the calciner (operating at 920–950 °C), the sorbent regeneration by the reverse endothermic process occurs. The reaction, sustained by oxy-combustion of Carbon-based fuel, generates a gas stream of nearly pure CO_2_, ready for sequestration after proper purification [15].

**Figure 2:**
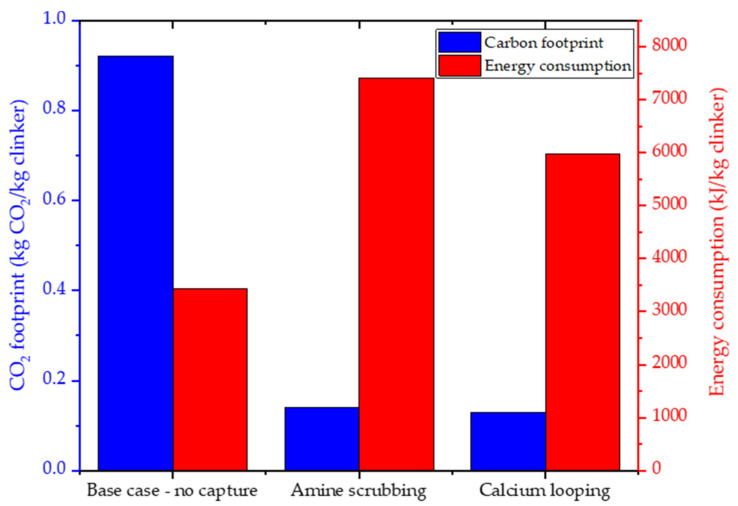
Efficiency analysis between CO_2_-capturing technologies. Reprinted with permission from ref. [16]. Copyright 2021 Elsevier.

The technical performances of the Ca-looping process were well investigated by Vatopoulos and Tzimas [16]. In particular, the authors propose a modelling analysis on the efficiency of CO_2_ capture options that can be employed in the short and medium term to the existing or new cement plants. Figure 2 reports the salient results of the comparative study, which highlights the performances of capturing technologies in terms of CO_2_ emission (kg/kg clinker) and overall energy consumption (kJ/kg clinker). 

The proposed capture technologies allow reducing CO_2_ emission by 84% with respect to the base system (no CO_2_-capturing technology). The increase in energy consumption (45% for amine scrubbing and 18% for Ca looping) is attributable to the greater energy requirement for the CO_2_ purification and compression system and the additional fuel required to operate the calciner.

### 2.3. Alternative Cementitious Binders

Calcium aluminates cements (CACs), super sulfated slag cements (SSCs), microbial cements (MCs), and geopolymer cements (GCs) are some types of sustainable binders as an alternative to conventional PC. Promising environmental performances are demonstrated by the production and adoption of these alternative materials: 5–90% reduction in CO_2_ release could lead up to 7% decrease in global CO_2_ emissions compared to PC manufacturing [17]. Coupled with the interest in finding solutions with low-CO_2_ emissions and low-energy consumption, the development of “green” binders aims to enhance the recycling and reuse of waste materials. In PC technology, some types of industrial waste (fly ash, silica fume, ground granulated blast furnace slag) are only partially used as supplementary cementing materials (10–50%). Nowadays, the research pushes beyond this evidence to create alternative cementitious compounds made entirely or almost entirely from waste products [18]. Besides, further motivation for exploring novel materials to supplement and/or replace PC concrete in the building architectural field derives from their technological performance. Specifically, many of these alternative and novel binder systems generally demonstrate increased durability when subjected to harsh conditions (such as lower shrinkage, higher acid resistance, fire inertia) compared to PC concrete [18]. However, one of the most relevant obstacles to the application of non-conventional cements concerns the lack of official standards or regulations unanimously accepted by the technical committees [18,19]. Next is an overview of the alternative cementitious binders mentioned above.

#### 2.3.1. CACs

CACs were developed in 1913 by Lafarge company (France). Originally, the growing interest in these cementitious compounds was related to their rapid hardening properties and good durability in severe environments [17,20]. Calcium aluminates are the main phase constituents: 10–40% Tetracalcium alumino ferrite (4CaO∙Al_2_O_3_∙Fe_2_O_3_) and 40–50% Monocalcium aluminate (CaAl_2_O_4_). CaCO_3_ and other impurities are secondary components. The raw materials, CaCO_3_ and bauxite (Al_2_H_2_O_4_) as Calcium (Ca) and Aluminum (Al) sources, respectively, are calcinated around 1450 °C to form Calcium aluminate phases. The hydration process leads to the generation of hexagonal Calcium-alumino hydrates (CaAl_2_O_4_·10H_2_O and Ca_2_Al_2_O_5_·8H_2_O). Hydrate products are metastables and can convert to stable phases by a thermodynamic stabilization process named “conversion”. When a conversion occurs, low-density Calcium-alumino hydrates convert into high density phases (3CaOAl_2_O_3_∙6H_2_O and Al_2_O_3_∙3H_2_O), releasing water and increasing the porosity in the hardened matrix, with a subsequent decrease in mechanical strength. The water-to-cement (*w/c*) ratio, humidity, and curing temperature are crucial variables for the quality and long-term durability of this cement [17,18,20]. 

Although this class of cementitious materials exhibits mechanical performance comparable to ultra-high-strength concrete (compressive strength over 150 MPa), the employment in structural applications is severely limited by its high sensitivity to environmental conditions, resulting from phase conversion described above [21]. Moreover, CACs are 4–5 times more expensive than Portland cements and, therefore, do not compete in any application where conventional concrete performs satisfactorily [20]. The major field of application of CACs are [17,20,22]:

- Refractory materials for industrial use in high-temperature processes.
- Repair material for infrastructural applications and protective coatings due to excellent resistance to chemical attack and abrasion.
- Printable materials in additive manufacturing (AM) technologies due to fast setting after deposition.

#### 2.3.2. SSCs

Pioneering studies on SSCs were conducted by Kuhl in 1909 [18]. Normally, a SSC mixture consists of 80–85% ground granulated blast furnace slag, 10–15% sulfate activator (anhydrite or gypsum), and a low amount (about 5%%) of alkali activator (clinker or cement) [23]. The hydration products, namely “slag gel”, are substantially the same as those identified in hydrating PC paste (e.g., Calcium-silicate hydrate and ettringite), with the additional presence of a hydrotalcite-like phase (Mg_6_Al_2_CO_3_(OH)_16_·4(H_2_O)) [24]. Such combination of mineral phases allows lower heat of hydration than common PC (90 J/g in SSCs manufacturing [18] against 235 J/g in PC production [15]), resulting in a lower contribution of thermal cracking phenomena. This peculiarity confers the good structural compaction, chemical durability, and frost resistance of the material. Besides, the low content of cement, and the main use of gypsum and slag, indicate a reduced energy impact related to clinkering and a considerable consumption of solid wastes [17,18,25]. The type and fineness of the slags play a key role in the rheological and strength properties of sulfated-based binders. Particles with a large surface (>500 m^2^/kg) area and sharp morphology reduce the workability and the mechanical strength of SSCs [17,23]. Besides, their contribution affects the setting time of the compound (1 h to 10 h), which is longer than for PC [18].

The marked durability in chemically aggressive environments makes these alternative binders suitable for structural applications (plasters or masonry mortars) in chemical processing plants or seawater civil projects [17]. The sustainability and economic impact of SSC technology is dependent on the sourcing of raw materials. In recent years, the supply of blast furnace slag has proved problematic due to the closure/inactivity of blast furnaces and depletion of old piles of waste, resulting in high slag demand and the growing cost of aluminosilicate materials [21]. In this regard, alternative raw material sources (such as pozzolanic volcanic glassy rocks, fly ash, flue gas desulfurization gypsum) are being explored [23,26].

#### 2.3.3. MCs

Extensive research on MCs was performed by Rong in the last decade [27]. MCs are a new generation of cements formed by a complex cementation mechanism based on microbiologically induced precipitation of CaCO_3_, named “biomineralization”. Three components are involved in the production process of MCs: Ca ion solution, a substrate solution (typically urea solution), and alkaliphilic microorganisms. Biomineralization is basically developed in four steps [27,28]:

- *Urea hydrolysis*. In a Ca-rich environment and an aqueous medium, a substrate solution is broken down into Ammonium (NH_4_^+^) and Carbonate (CO_3_^2−^) by the bacterial enzyme (microbial urease), according to the following reaction:
  $$\mathrm{Urea}\;\mathrm{substrate}+2H_{2}O\overset{\mathrm{bacteria}}{\to }{\mathrm{CO}}_{3}^{2-}+2{\mathrm{NH}}_{4}^{+}$$
- *Calcite precipitation.* Due to the breakdown of the substrate, the pH value around the surrounding environment increases. The presence of Ca ion results in CO_3_^2−^ precipitate as CaCO_3_ crystals.
  $${\mathrm{Ca}}^{2+}{\mathrm{CO}}_{3}^{2-}\to {\mathrm{CaCO}}_{3}↓$$
- *Ca-microorganism coordination*. In addition to calcite precipitation, due to the complementary net charge, positive Ca ions deposit on the negative microorganism surface, generating nucleation sites on the cell wall.
  $$\mathrm{Cell}-{\mathrm{Ca}}^{2+}+{\mathrm{CO}}_{3}^{2-}\to \mathrm{Cell}-{\mathrm{CaCO}}_{3}↓$$
- *Generation of cementitious mass.* CaCO_3_ crystals generate cementitious connections between the mineral aggregates. The presence of calcite sites protects the microorganism from the highly alkaline concrete environment and promotes the diffusion of the cementitious bridge until obtaining a single mass.

The biomineralization mechanism is a very attractive solution to enhance the self-healing properties of cementitious materials. When the material is damaged, water from in the outside ambient will penetrate through the structural cracks. Inside the cement matrix, favorable conditions for biologically induced precipitation are created. Then microorganism will activate the biomineralization process, generating CaCO_3_ precipitates which will solidify on the damaged surface, sealing the material. Furthermore, during their activity, the bacteria consume oxygen avoiding corrosion phenomena and increasing the concrete durability [29]. In terms of mechanical behavior, Thakur et al. [30] revealed an enhanced mechanical strength in bacteria-based concrete than Portland control samples. As shown in Table 1, the type of bacterial species is a key factor in the strength increase rate due to the different metabolic activity taking place during biomineralization. 

Bacterial concentration is a further variable to consider for reaching optimal structural and durability properties in MC-based material. As reported by Mondal and Ghosh [35], for each bacterial species, the best concentration to obtain relevant improvement in mechanical strength lies between 10^5^–10^7^ cells/mL, while self-healing behavior is promoted for higher concentrations (10^8^–10^9^ cells/mL). However, increasing the bacterial level decreases the mechanical performance of the material, according to mechanisms still unknown. Current studies aim at the investigation of optimal bacterial concentration for the enhancement of the engineering properties of microbial concrete.

Despite the interesting peculiarities of this alternative binder (CO_2_-emissions free technology, self-healing capability, strength improvement,), numerous aspects limit its large-scale manufacturing [29]:

- *High cost of bacterial cultures*. The overall price of MC products is up to four times higher than conventional PC concrete. Besides, the manipulation of bacterial species requires skilled personnel, which increases the cost of R&D activities.
- *Sensitivity of biological raw materials*. MCs performance may significantly vary with geographic and environmental location. Besides, bacteria are considered dangerous to health and strict controls are required.
- *Lack of standard*. Nowadays, there are still no technical recommendations or protocols in place concerning the testing and acceptance criteria.
- *Long-term durability*. MCs are an emerging technology, and experimental research or analysis on the long-term (at least 50 years, as suggested by technical standards) durability and anti-corrosiveness of the bacterial-based cement products are not available.

## 3. Geopolymer Cements: Basic Features and Novel Advances

**Figure 3:**
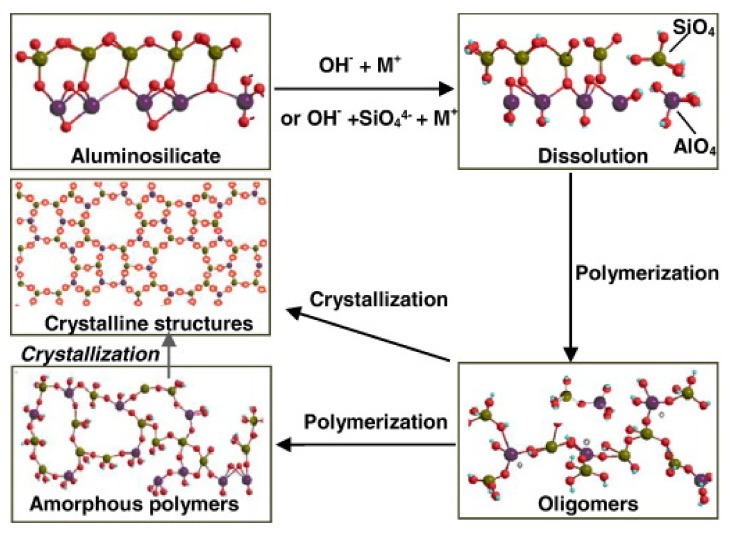
Zhang model of the geopolymerization process. Reprinted with permission from ref. [40]. Copyright 2016 Elsevier.

Geopolymer cements result from a chemical reaction between aluminosilicate materials and alkali solution. “Cement” does not mean the chemical characteristics of the material but rather its functionality as a matrix applicable in the field of concrete materials. Specifically, the precursors are rich in SiO_2_ and Al_2_O_3_. When activated by alkali solution, these materials lead the formation of an aluminosilicate gel that develops high strength at early age and after moderate curing temperature condition [36]. Based on the nature and type of the aluminosilicate precursors, geopolymer binders can be mainly classified in [37]: granulated blast furnace slag (GBFS)-based geopolymer cement, fly ash (FA)-based geopolymer cement, rock-based geopolymer cement, and Ferro-sialate-based geopolymer cement. These binders comprised of a repeating unit of silico-oxide (–Si–O–Si-O–), silico-aluminate (–Si–O–Al–O–), Ferro-silico-aluminate (–Fe–O–Si–O–Al–O–) or alumino-phosphate (–Al–O–P–O–), developed through an alkali-activated geopolymerization process [38]. Alkaline compounds, such as Sodium hydroxide (NaOH), Potassium hydroxide (KOH), Sodium silicate (Na_2_SiO_3_), and Potassium silicate (K_2_SiO_3_), are used to activate the aluminosilicate precursors. The reaction produces SiO_4_ and AlO_4_, tetrahedral frameworks linked by shared oxygen as poly(sialates) or poly(sialate–siloxo) or poly(sialate–disiloxo), depending on the SiO_2_/Al_2_O_3_ ratio in the system. The tetrahedral units are alternatively linked to polymeric precursors by sharing oxygen atoms, thus forming polymeric Si–O–Al–O bonds [39]. A comprehensive geopolymerization mechanism was proposed by Zhang et al. [40] and reported in Figure 3. Synthetically, the mechanism involves the following steps: (a) dissolution of aluminosilicate particles; (b) initial polymerization of dissolved alumina and silicate species into Al–O–Si oligomers; (c) further polymerization into large amorphous gels (zeolitic precursors) or direct growth into crystalline structures (zeolitic phase). After the gel is formed, the system will continue to rearrange and the number of bonds between the molecules will also increase, creating a 3D network that is associated with the geopolymer structure.

The geopolymer synthesis and structure are very similar to zeolites. The main difference is related to the crystallinity degree. Zeolite is usually crystalline in nature. It crystallizes from dilute aqueous solution by hydrothermal process, where aluminosilicate precursors have enough mobility and time to orient and align before bonding into a crystal lattice. Conversely, geopolymers are amorphous to semi-crystalline. During the alkali activation of precursors, there is not sufficient time and space for the gel to arrange into a crystalline framework. The result is an amorphous or semi-amorphous C–S–H microstructure [36]. 

The preparation of geopolymer materials, including concrete and mortars, is summarized in the following steps [41]:

- *Preparation of the activator solution.* The alkali solution is prepared at least one day prior to its use, employing distilled water to avoid contamination by unknown substances. Common molarity values investigated are in the range of 8 to 16 M.
- *Raw material mixing.* One or more precursor types are mixed with the mineral aggregates (fine sand or coarse fraction), in dry conditions, for 2–3 min. The aggregate part occupies about 75–80% by mass of geopolymer concrete/mortar.
- *Activator addition.* The alkaline solution is added to the solid fractions and mixed for 3–5 min. To improve the rheology of the compound, in terms of workability, a part of water-reducing admixture could be added to the mixture. Firstly, the admixture is mixed with the alkali solution and then added to the solid dry material.
- *Casting.* The fresh material is cast in specific molds (cylinder, cubes, or beams) kept under vibration for 10 s.
- *Curing.* The specimen curing can be performed under various temperature and time regimes. The material hardening can be completed entirely at room temperature or provide oven treatments between 30 °C to 90 °C, accelerating the curing. Typical treatment times are selected in the range from 6 h to 96 h.

As clearly discussed above, the production of geopolymer cements involves a considerable number of process variables. The microstructure and the physical, mechanical, chemical, and thermal properties vary greatly depending on the nature of raw material from which they derive, the curing conditions (time and temperature), and the type and concentration of the activating alkaline solution [42]. In the next sections, some salient results of research focused on the influence of the synthesis parameters on the final properties of geopolymer binders will be reported.

### 3.1. Influence on Mechanical Strength Performance

Below, a series of studies are collected and reported to evaluate how process parameters (curing regime, characteristics of precursors, molarity of the activating solution, Si/Al elemental ratios) affect the mechanical and structural performance of geopolymer-based materials. 

Aredes et al. [43] investigated the effect of curing temperature on the strength performance and microstructural characteristics of MK-based geopolymer. Keeping the sample formulation fixed (MK and amorphous silica with a mass ratio of 0.2, K_2_O_3_Si activating solution with pH 14, 11.5 *w*/*w*% of extra water), three curing regimes were investigated: 55 °C, 65 °C, and 80 °C for 1 h. The maximum strength (12 MPa) was obtained at 65 °C. The lowest values were recorded at 55 °C and 80 °C, where the range of compressive strengths were 6.9 MPa and 7.4 MPa, respectively. According to the authors’ discussion, the lowest temperature under study (55 °C) did not allow a proper geopolymerization degree in terms of complete activation of the aluminosilicate precursors. Unreacted particles act as mechanical defects in the structure, decreasing the strength of the material. Higher curing temperature (80 °C) led to a greater rate of open porosity (~30%) and macropores from water evaporation, which negatively affected the mechanical properties. Curing at 65 °C was the optimal condition in terms of microstructural quality. 

Oderji et al. [44] researched the effect of GBFS content and activator dosage (Na_2_SiO_3_) on the mechanical properties of FA-based geopolymer cements. The compressive strength increased significantly between 10% and 20% GBFS replacement of FA, reaching a value around 40 MPa at 28 days (curing temperature of 23 °C and 8% by mass of precursors of Na_2_SiO_3_). The increase in GBFS content indicated additional CaO-rich sources, which promote the development of the aluminosilicate gel following the alkali activation. By analysing the influence of a lower dosage of alkali activator (6% and 7%), a remarkable loss in strength was detected. A strength reduction of about 80% was found in the formulations with 6% and 7% (~5 MPa strength) of activator dosage with respect to the optimum Na_2_SiO_3_ amount of 8%. In this case, the change in Na_2_O adversely affected the strength gain in the material through an alkali activation reaction.

**Figure 4:**
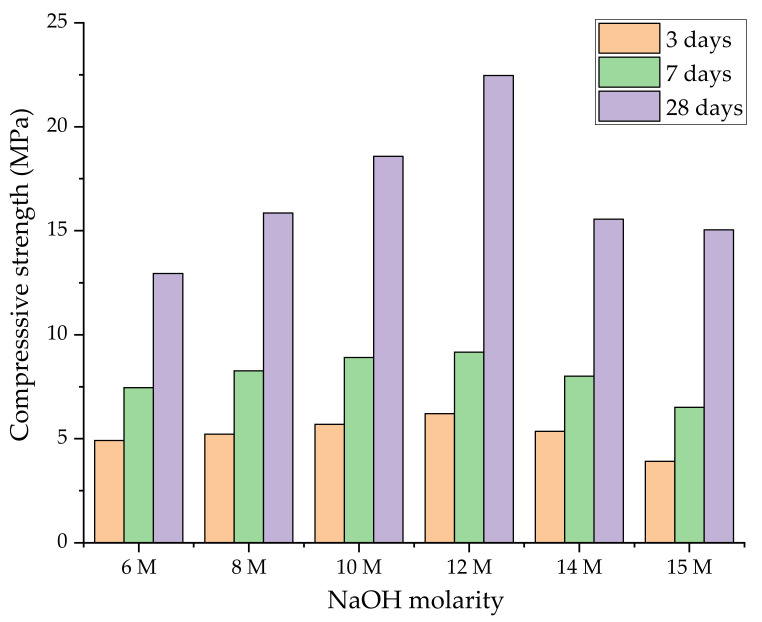
Effect of activating solution molarity on strength performance of FA geopolymer [45].

Concerning the alkali solution molarity, Wardhono [45] studied the mechanical performance of FA-based geopolymers by varying the concentration of NaOH activating solution (6 M, 8 M, 10 M, 12 M, 14 M, and 15 M). The strength properties evaluated at 3, 7, and 28 days (after ambient curing) are presented in Figure 4. As expected, regardless of the molarity of the activator, all mixtures gained mechanical strength as the cure time advanced from 3 to 28 days. NaOH at 12 M appeared to be the best condition in terms of strength properties (about 22.5 MPa). An increase in molarity indicates an acceleration of the activation process due to favourable dissolution conditions for the aluminosilicate precursors. Above this optimal threshold value, a gradual fall in mechanical properties was noted, making the role of the solution on the alkali activation of precursors ineffective. The major strength reduction (around –33%) was found in the sample activated with 15 M solution. 

**Figure 5:**
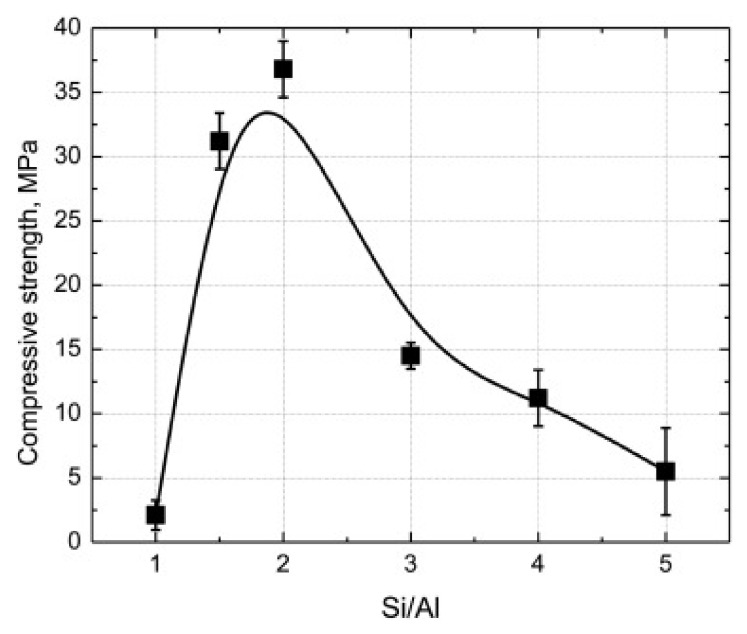
Relationship between strength and Si/Al ration in MK-based geopolymers. Reprinted with permission from ref. [46]. Copyright 2017 Elsevier.

Wan et al. [46] determined a relationship between the mechanical strength of MK-based geopolymer binders and Si/Al ratio. To study various Si/Al levels (1:1, 1.5:1, 2:1, 3:1, 4:1, and 5:1), six corresponding geopolymer formulations at different dosages of Silica fume (0 mol, 0.5 mol, 1 mol, 2 mol, 3 mol, and 4 mol, respectively) were prepared and characterized. The 7 days compressive strengths (after 6 h of curing treatment at 60 °C) as a function of Si/Al ratio are plotted in Figure 5. Compressive strength increased from 2.1 MPa (Si:Al ratio of 1:1) to the maximum value of 36.8 MPa (Si:Al ratio of 2:1). Once this Si:Al proportion was exceeded, a gradual decrease in mechanical strength was recorded reaching a value of 5.5 MPa for Si:Al ratio of 5:1. This trend was attributed to the proper development of the aluminosilicate gel. For Si:Al ratio of 1:1, geopolymer presented a Zeolite-based crystalline pattern with a reduced geopolymeric phase. Si:Al ratio of 2:1 was the better proportion to achieve a good quality aluminosilicate gel from the adequate dissolution of the precursors. Excessive silicate dosage weakened the geopolymer’s strength due to the insufficient amount of dissolved aluminate and silicate monomers to form a well-structured binder.

Selecting the Si:Al ratio, it is possible to define specific microstructural and technological properties of the geopolymers. Low Si:Al ratios (until 3:1) give a very rigid 3D framework, while Si:Al ratio higher than 15:1 provides a polymeric character to the binder. This evidence makes the geopolymer a very versatile technology in the civil industry [47]. Based on Si:Al atomic ratio, it is possible to identify a wide range of applications (Table 2).

### 3.2. Influence on Microstructural Features 

**Figure 6:**
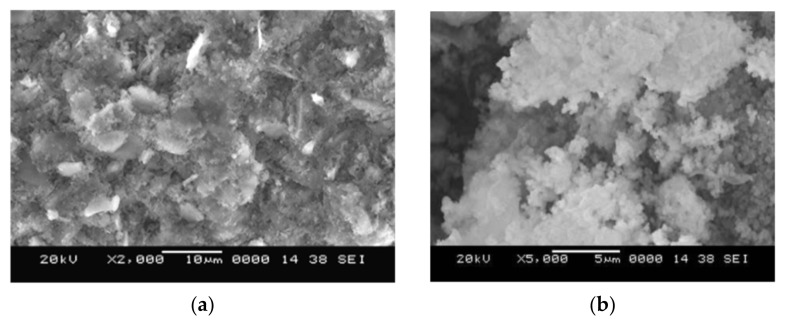
SEM analysis of geopolymer matrices with FA (**a**) and MK (**b**). Reproduced with permission from [48]. Copyright 2013 Scientific.Net.

Figure 6 below shows the detailed scanning electron microscopy (SEM)-determined microstructure of FA and MK as different aluminosilicate precursors used in the preparation of the Geopolymer cement. 

FAs in the geopolymer microstructure (Figure 6a) appear as spherical, glassy, and hollow particles. In the matrix, the coexistence of these precursors with iron and mullite crystals can be detected. FAs provide a higher Si/Al level than MK precursors, resulting in a more heterogeneous microstructure due to the presence of unreacted particles. In addition, FA-based geopolymer contains pores in the micropore size range. On the other hand, MK-based geopolymer (Figure 6b) presents a “sponge”-like gel, indicating a more effective alkaline activation of precursors. MK geopolymers contain air voids that are predominantly in the mesopore size range [48]. 

**Figure 7:**
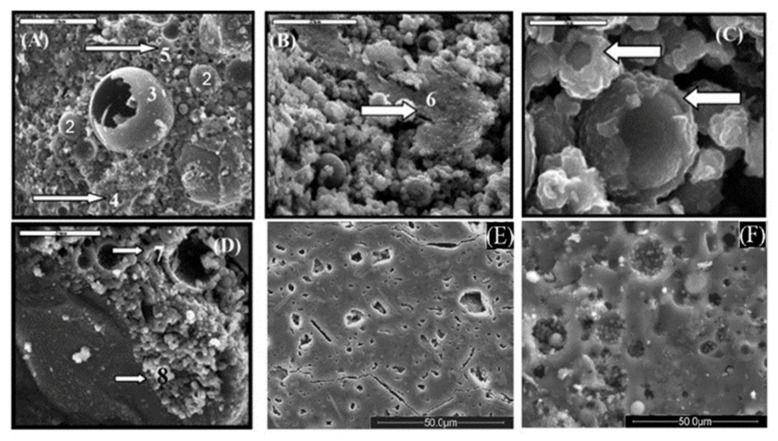
SEM micrographs of FA and MK-based geopolymers: FA morphology (**A**), zeolitic crystalline phase on the source materials (**B**,**D**), FA particles not completely covered by the reaction product (**C**), microstructures of MK-based geopolymer (**E**), and microstructure of FA-based geopolymer (**F**). Reprinted with permission from Ref [49]. Copyright 2016 IOP Publishing.

A similar comparative investigation between FA and MK-based geopolymer microstructures was performed by Nuruddin et al. [49]. Figure 7A–D shows SEM images for FA-based geopolymer. These micrographs show that the main component of the FA-based geopolymer is aluminosilicate gel (tags 4 and 5). It is possible to observe a large amount of rounded, unreacted, or incompletely reacted FA particles (respectively in tags 2 and 3). Among the spherical particles of various sizes in the range of 10–200 μm, solid and hollow spheres (tag 3, Figure 7A) are detectable. The image also shows the generation of a zeolitic crystalline phase. However, some of these crystals originally existed in raw materials, with quartz and hematite as the crystalline phase. These crystals are represented by tags 6, 7, and 8 in Figure 7B,D. Figure 7C shows that some FA particles are not completely covered by the reaction product, indicating an incomplete or weak geopolymerization process. Figure 7E,F shows the microstructures of MK and FA-based geopolymers, respectively. It should be noted that the degree of reaction mainly depends on the form of the raw material. MK particles are in the form of layered sheets. Compared with spherical FA particles, the dissolution reaction of MK particles makes the surface layer peel off, while with FA, the dissolution reaction products deposit on the outer surface. This will cause the additional layer of MK particles to react, and the reaction will continue. However, in the case of FA particles, the precipitated product encapsulates the surface of the particles and prevents further dissolution, during which time the reaction is diffusion controlled. This slows down the reaction rate and more particles remain unreacted or partially reacted.

**Figure 8:**
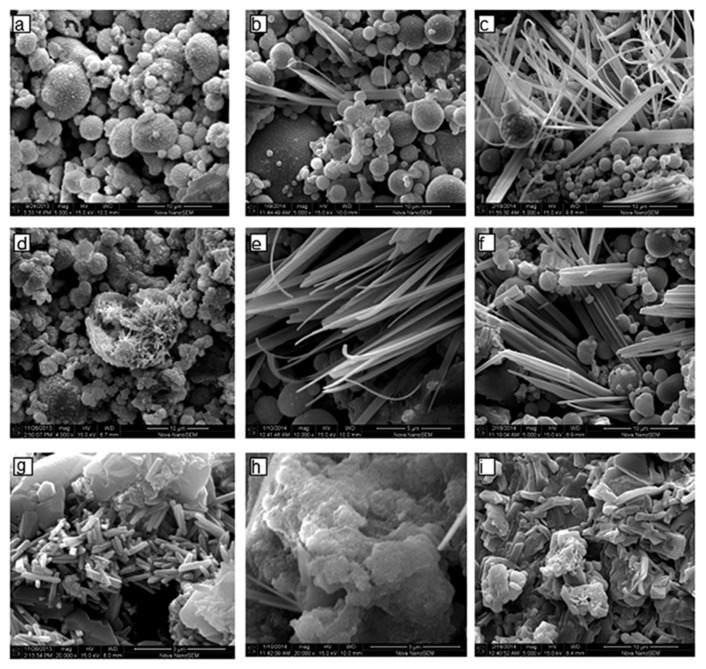
SEM pictures of FA-based geopolymers considering different process conditions (alkali solution molarity and curing temperature): (**a**) 6 M—27 °C; (**b**) 6 M—45 °C; (**c**) 6 M—60 °C; (**d**) 8 M—27 °C; (**e**) 8 M—45 °C; (**f**) 8 M—60 °C; (**g**) 10 M—27 °C; (**h**) 10 M—45 °C; and (**i**) 10 M 60 °C. Reprinted with permission from Ref. [50]. Copyright 2016 Elsevier.

Nath et al. [50] presented a detailed SEM analysis on the microstructure evolution of FA-based geopolymer binders following the change in alkali concentration and curing temperature. The micrographs are reported in Figure 8.

The observation from SEM investigations combined with energy dispersive X-ray spectrometry (EDS)-based elemental analysis are summarized in Table 3.

### 3.3. Influence on Mineralogical Characteristics

**Figure 9:**
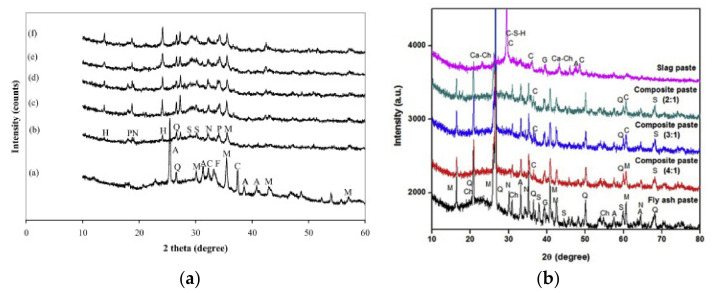
XRD characterization of FA-based geopolymer binders: (**a**) Effect of activator molarity. Reprinted with permission from Ref. [51]. Copyright 2014 Elsevier. and (**b**) Influence of GBFS content. Reprinted with permission from Ref. [52]. Copyright 2019 Elsevier.

Hanjitsuwan et al. [51] studied, by X-ray diffraction (XRD) technique, the crystallography of FA-based geopolymer paste with various activator (NaOH) concentrations (8 M, 10 M, 12 M, 15 M, 18 M). Figure 9a (label “a”) reports the patterns of unbonded FA particles, consisting of two phases: an amorphous phase indicated by the broad peak at 20–38° and a crystalline phase related to the sharp peak of mineralogical components such as SiO_2,_ CaO, and hematite (Fe_2_O_3_). The alkali activation of FA formed a new phase composed by geopolymeric and Calcium silicate hydrate (CSH) gel, shifting the peak around 25–38°. With the increase in NaOH concentration, CSH intensity peak increases, resulting in a better dissolution process of the precursors and higher strength of the geopolymer paste. Ishwarya et al. [52] monitored the mineralogical features of binary geopolymeric systems (FA-GBFS) by considering three different FA:GBFS ratios (4:1, 3:1, 2:1). As shown in Figure 9b, geopolymer composites showed a diffuse broad peak in the range of 16–39°, while the inactivated precursors provided hump at 15–30° (for FA) and 23–40° (for GBFS). The peak shifting in the geopolymer samples was attributed to the formation of amorphous NASH (Na-silico-aluminate hydrate) gel, resulting from the activation of aluminosilicate precursors. By increasing the slag content, the area of these peaks increased, indicating a higher amount of gel in the matrix and greater strength.

**Figure 10:**
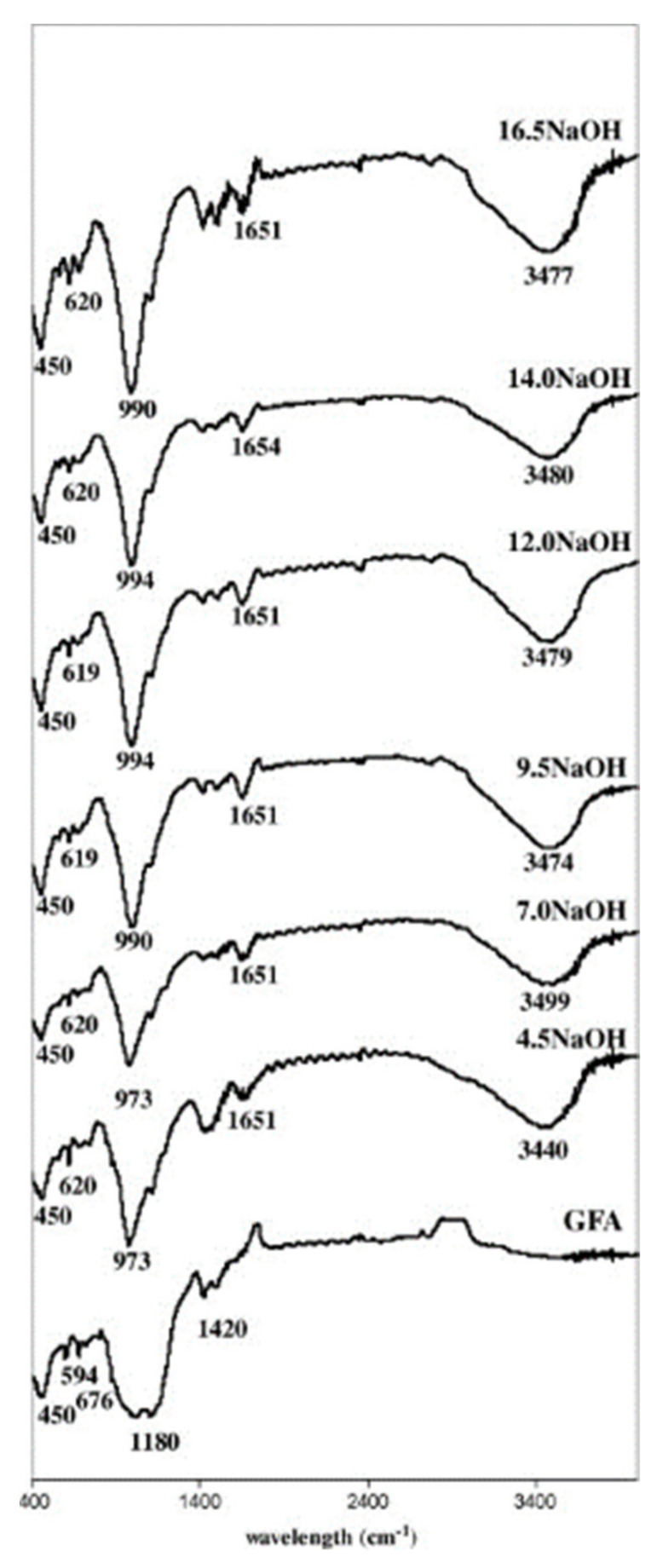
FT-IR spectra of FA-based geopolymer cement: influence of molarity solution. Reprinted with permission from Ref. [53]. Copyright 2014 Elsevier.

Fourier-transform infrared (FT-IR) spectroscopy was employed by Somna et al. [53] to evaluate the molecular fingerprint of FA-based geopolymer binders activated with different alkali concentrations (from 4.5 M to 16.5 M). By comparing the infrared spectrum of FA with that of the geopolymer samples, four fundamental bands can be identified (Figure 10):

- 450 cm^−1^ bands associated with Si–O–Si bending vibration and common to both the precursor and geopolymer samples.
- A peak around 900–1200 cm^−1^ associated with Si–O–Si and Si–O–Al asymmetric stretching vibration. This peak shifts to shorter wavelengths in geopolymer samples than FA as a result of the activation process and different Si/Al ratio in the system.
- Broad bands around 1650 cm^−1^ and 3480 cm^−1^ assigned to water (–OH stretching vibration and O–H–O bending vibration, respectively. These peaks are not detectable in the FA sample, indicating the advent of geopolymerization process.

The effect of Si/Al ratio on FT-IR spectra of MK-based geopolymer cement was evaluated by Ozer et al. [54]. By analyzing three different Si/Al levels (1.12, 1.77, and 2.20), the authors demonstrated that the band located around 1000 cm^−1^ (asymmetric stretching vibrations of Si–O–Si and Si–O–Al) is mainly subject to shifting as the aluminosilicate ratio varies. This band systemically shifts to higher wavenumbers and its intensity decreases with increasing Si/Al ratio. This effect is attributed to higher geopolymerization degree and disorder in the system.

### 3.4. Influence on Porosity and Implications on Strength Properties

**Figure 11:**
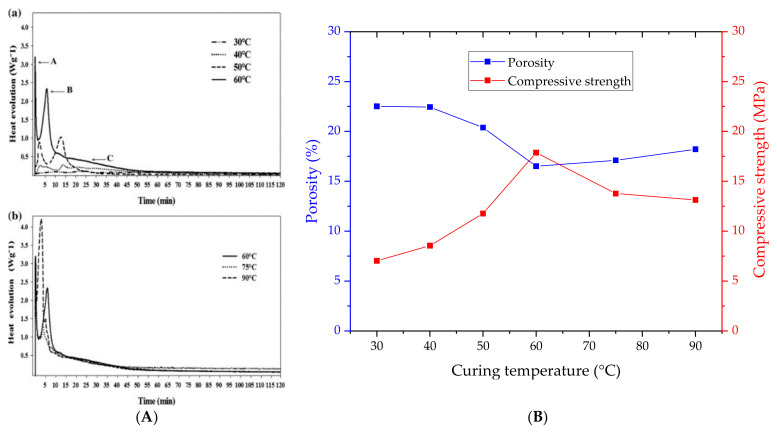
(**A**) DSC thermograms of MK-based geopolymer cement as a function of curing temperature: (**a**) 30 °C, 40 °C, 50 °C, and 60 °C and (**b**) 60 °C, 75 °C, and 90 °C. (**B**) Effect of curing regimes on porosity and mechanical strength. Reprinted with permission from Ref. [55]. Copyright 2011 Elsevier.

By differential scanning calorimetry (DSC) measurement, Muniz-Villareal et al. [55] studied the effect of six curing temperatures (30 °C, 40 °C, 50 °C, 60 °C, 75 °C, and 90 °C) on the geopolymerization process of MK-based geopolymer cements. The heat evolution plots (Figure 11A, tags “a” and “b”) reported three exothermic peaks related to the following phenomena: the dissolution of the solid precursors in the strong alkaline solution (peak “A”), the formation of aluminosilicate oligomeric species in the solution (peak “B”), and the polymerization/condensation reactions until the consolidation of the geopolymeric network (peak “C”). By comparing the thermograms at different curing regimes, it was observed that low temperatures (from 30 °C to 50 °C) implied incomplete and inefficient dissolution of the precursors and subsequent formation of oligomers. Too high temperatures (75 °C and 90 °C) resulted in shorter times for the proper activation of the precursors. Furthermore, in this last case, the rapid evaporation of water was promoted, leading to the formation of microcavities and an increase in porosity. The curing at 60 °C provided the favorable condition for the geopolymerization, giving the best results in terms of porosity degree and mechanical strength (Figure 11B). 

**Figure 12:**
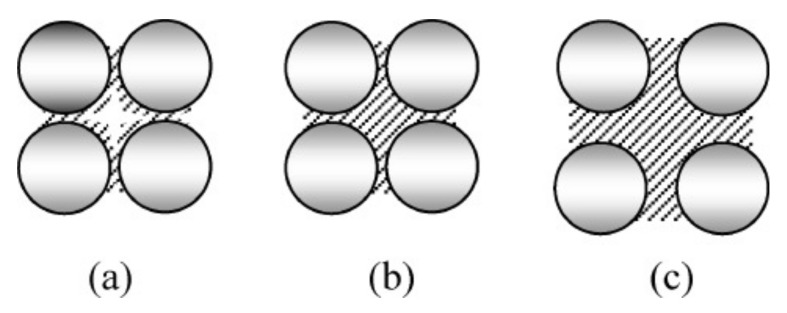
Schematic illustration of the effect of increasing molarity levels on the formation of geopolymeric binder around FA particles, in accordance with the mechanism proposed by Fansuri et al.: (**a**) insufficient amount of binder that incorporates FA particles due to low molarity condition, (**b**) optimal condition in terms of alkaline activator molarity and microstructural quality, and (**c**) excessive molarity level and consequent inhibition of the precursor activation. Reprinted with permission from Ref. [56]. Copyright 2010 Wiley.

Fansuri et al. [56] proposed a mechanism to describe the influence of NaOH-activator molarity on the dissolution of FA precursor and development of geopolymer paste. In the alkali activation of FAs, the solution molarity plays a crucial role in the release of silicate and aluminate monomers and the consequent formation of the geopolymeric gel. According to the authors’ findings, the concentration should be correctly dosed to obtain a proper dissolution process and optimal achievement of geopolymer phase, which acts as a binder for the unreacted precursors. The effect of increasing molarity on FA activation is illustrated in Figure 12.

Too low levels of molarity (Figure 12a) result in a large amount of unreacted FA particles surrounded by a matrix with many empty spaces due to the insufficient amount of binder that is formed following alkali activation. Excessive alkali conditions (Figure 12c) imply a more effective dissolution of precursors and the development of a large amount of geopolymer gel surrounds the few non-activated FAs. However, such a situation is not ideal as the strength of the material will be controlled almost entirely by the properties of the geopolymer matrix, which has a lower mechanical strength than the precursors (~10 MPa for geopolymer paste and more than 1 GPa for quartz-based precursors). The optimum condition is presented in Figure 12b, where the geopolymeric gel will occur in optimal quantity to ensure the proper bond between unreacted particles, avoiding voids and microstructural defects. Furthermore, the influence of precursors will relevantly contribute to the strength performance, acting effectively as reinforcement fillers. Confirming the mechanism described above, Table 4 reports some literature results about optimal molarity values in terms of impact on porosity degree and mechanical strength.

Another noteworthy process variable to investigate the impact on porosity and strength of geopolymer binders is the initial amount of water, which is related to the preparation of the activating solution. Pouhet et al. [60] prepared five MK-based geopolymer formulations at different water/solid mass ratios: 0.38, 0.40, 0.50, 0.60, and 0.70. Increasing the water content decreased the mechanical performance. The 7 days compressive strength falls linearly from 65.6 MPa for 0.38 ratio to 3.0 MPa for 0.70 ratio. This behavior is different from the non-linear decay found in ordinary cements as the water dosage increases. Indeed, in geopolymeric compounds, water does not participate in the hydration of the binder and therefore in the structuration of the binder matrix, but it affects the pore structure of the material, weakening its mechanical properties. In this context, the authors found a direct proportionality between the water introduced and the final porosity. For the water/solid ratio around 0.60, a porosity value greater than 55% was detected. Similar results were observed by Aliabdo et al. [58]. Here, the effect of four additional water levels (10 kg/m^3^, 20 kg/m^3^, 30 kg/m^3^, and 35 kg/m^3^) in the FA-based geopolymer mix design was investigated. The gradual increase in additional water increases the material porosity, which ranged from 9.8% to 11.1%. The growth in porosity was accompanied by a drop in 28 days compressive strength, which ranged from more than 35 MPa (water addition of 10 kg/m^3^) to about 25 MPa (water addition of 35 kg/m^3^).

### 3.5. Influence of Nanostructured Materials on Geopolymer Performances

One of the most recent and advanced upgrades of geopolymer technology concerns the functionalization with nanomaterials, identified as performance enhancers to improve the microstructure, rheology, and the hardened state properties of alkali-activated composites. By exploring the current literature, it emerged that various types of nanostructured materials (nanoparticles, nanofibers, and nanostructured sheet) were employed to optimize the geopolymer mix designs. A collection of the most relevant studies on the nanoengineering of GPCs as a function of the nanofillers’ dimensionality is reported below.

#### 3.5.1. Nanoparticles

Assaedi et al. [61] investigated the influence of Calcium carbonate nanoparticles (nano-CaCO_3_) on the microstructure and mechanical properties of geopolymer composite developed from activation of FA precursors. The authors identified that an additional level within 3 wt% allows nanofillers to provide significant strengthening effect and denser binding matrix. Nano-CaCO_3_ act as nucleation sites for additional CSH-based hydration product’s development, as well as perform a filling effect for geopolymer paste’s microstructure. For 3 wt% of nano-CaCO_3_ content, 38% increase in compressive strength and 55% increase in flexural strength were detected.

Maiti et al. [62] studied the incorporation of rutile-phase Titanium oxide nanoparticles (nano-TiO_2_) in FA-based GPC, investigating the mechanical strength and durability performance as a function of nanoparticles size (30 nm, 50 nm, and 100 nm). At fixed amount of nano-TiO_2_ (5 wt%), the experimental results revealed gradual increments in compressive strength in geopolymer nanocomposites compared to unmodified mix. The high surface area of nano-TiO_2_ accelerated the geopolymerization and development of aluminosilicate gel. Maximum strength increase (26.5%) was found in 30 nm-size nanoparticles due to the better effect of finest fraction to fill up voids and pores in geopolymer paste. Consequent to the improved matrix densification, superior performance was found in terms of water permeability resistance, indicating nano-TiO_2_ as effective fillers regarding the material durability and anticorrosion properties. Water absorption passed from 15.90% in control mix (0 wt% of nano-TiO_2_) to 11.07% (nano-TiO_2_ of 30 nm).

Mahboubi et al. [63] evaluated the influence of nano-clay (NC) powder and nano-silica (NS) particles on the mechanical strength and durability against acid attack of MK-based GPC. The nano-dimensionality of the two ceramic fillers stimulates their pozzolanic activity, thus improving the microstructural characteristics of the material. Moreover, the nano-sized particles exerted a filling effect for the pores and voids, reducing the permeable porosity of GPC. As a result of these effects, the authors reported that with the addition of 3 wt% of NC and NS, the 28 days compressive strength increased 1.44-times and 1.39-times, respectively, with respect to the plain mix. In terms of durability, under exposure to acidic medium for 28 days, the control sample suffered an average weight loss of 85%. The addition of the two nanofillers led to greater stability of the GPC with less significant losses (12% weight loss for 3 wt% of NC and 14% wight loss for 3 wt% of NS).

#### 3.5.2. Nanofibers

Akono [64] investigated the fracture behavior of MK-based GPC reinforced with Carbon nanofibers (nano-CF) in various levels (0.1 wt%, 0.2 wt%, and 0.5 wt%). By micro-indentation and microscopic scratch test, the author revealed ameliorative effects of nano-CF on the mechano-dynamic and elastoplastic properties of the material: (a) increase in indentation hardness of 9%, 18%, and 25%, respectively; (b) increase in fracture toughness of 38%, 40%, and 45%, respectively; and (c) increase in fracture energy of 83%, 72%, and 74%, respectively. Two fundamental mechanisms acted on the strengthening and toughening of the fiber-reinforced geopolymer composites: (a) the effect of nano-CF on the porosity refinement and material densification, and (b) crack bridging effect on the fracture behavior.

Rahman et al. [65] studied the influence of silicon carbide whisker (SCW) nano-fillers on the mechanical properties and morphology of geopolymer binder obtained from alkali activation of a binary precursor system (MK and rice husk ash). Addition of 4 wt% of SCW demonstrated a respective 97% and 158% increase in flexural and shear strength. High aspect ratio of nanofillers promotes the ability of the material to resist tensile failure by crack bridging mechanism. This factor is further promoted by the Si-rich composition of SCW fillers, which promotes a strong bond with the geopolymeric binder.

Alumina nanofibers (ANF) in FA-based geopolymer composites were investigated in [66]. The authors demonstrated that to obtain a functional strengthening effect, the fiber content must be carefully dosed. For a content of 5 wt%, an increase in the flexural strength occurred (11% increment with respect the reference mix), while further addition of nanofibers resulted in ineffective reinforcement effect due to segregation phenomena. ANF provided a beneficial impact on the high-temperature behavior of geopolymer mixes. High thermal resistance of alumina preserved the strength under exposure to high temperatures (>200 °C) by accommodating the shrinkage phenomenon during the heating process.

#### 3.5.3. Carbon-Based Nanostructured Sheet

Amri et al. [67] implemented FA-based GPC incorporating graphene nanosheets (GNs) in various concentrations of 5–20 mg/mL. As the GNs content increased, a progressive gain in compressive strength was recorded, reaching the maximum increment (around 114%) with a nanofillers addition of 20 mg/mL. The authors explained the effect of Carbon-based nanosheets on mechanical properties as follows. GNs established secondary chemical bonds with the oxygen atoms of the aluminosilicate chain (Si–O–Si or Si–O–Al) of the geopolymer matrix. Thanks to this cohesion, under compressive load conditions, the nanofillers ensured an effective stress distribution in the material, increasing its toughness at break.

Su et al. [68] reviewed the recent advances of geopolymer composites functionalized with Carbon nanotubes (CNTs). The main effects of these nanostructured materials on the properties of geopolymer compounds are listed below:

- *Fresh properties*. The high specific surface area of CNTs decreases the workability and increases the viscosity of the fresh mixes. However, insignificant change in rheology can be reached for low CNT concentration (less than 0.2 wt%).
- *Mechanical properties.* An incorporation of a very small amount of CNT leads a strong mechanical enhancement: 0.5 wt% of nano-sized fillers corresponded to a mechanical strength 110–130% higher than plain geopolymer mix. CNTs act as reinforcing fillers due to the excellent mechanical behavior and crack bridging properties. Besides, it was demonstrated that a proper dosage of CNTs decreases the total porosity of the material. However, there will be a limit concentration (also defined as a percolation threshold), depending on the initial property of CNT and mix design of geopolymer samples, above which agglomeration and poor dispersion of CNTs will occur, leading to strong losses in mechanical strength.
- *Durability.* Addition of CNTs below the percolation threshold reduces the overall porosity and improves the microstructural compaction of the material, minimizing its hygrometric shrinkage and permeability to water and other aggressive agents.
- *Smart properties.* The presence of CNT in the geopolymer matrix generates a conductive network which improves the electrical conductivity properties. This peculiarity can potentially be exploited in self-sensing application for structural health monitoring.

Long et al. [69] conducted a study on the reinforcing mechanism of reduced graphene oxide (rGO) nanosheets on GBFS-based geopolymer mortars. The addition of rGO nanosheets remarkably increased the flexural strength properties of the samples. In fact, the presence of these nanofillers provided many nucleation sites for the formation of aluminosilicate gel, accelerating the hydration reaction and making the matrix denser and less porous. Specifically, the authors identified a maximum strength increase of 51% when graphene oxide nanosheets were reduced at 60 °C. 

### 3.6. Section Summary 

In this section, the fundamental aspects of geopolymer technology in the context of concrete materials have been reviewed. The key feature of this new class of cements regards the strict relationship between synthesis parameters and technological performance of the final material. In this regard, the influence of the process variables, including curing regime, alkali activator molarity, type of aluminosilicate precursors, mechanical strength, mineralogical/microstructural characteristics, and porosity, was investigated considering more than 30 references from research literature. Finally, as an innovative upgrade of the technology, a collection of works based on the functionalization of geopolymer matrices with nanostructured fillers was presented, revealing a significant improvement impact on the performance of the material in terms of strength and durability.

## 4. Geopolymer vs. Ordinary PC Mixes: A Comparative Analysis

“*Can Geopolymers be competitive to PC in the civil and construction sectors?*” To evaluate the peculiarities of geopolymeric binders as a possible eco-friendly alternative to traditional cements, this section reports a collection of comparative analyses between geopolymer and Portland concrete materials, based on several technological and sustainability criteria.

### 4.1. Geopolymer vs. Ordinary PC Mixes: Chemistry and Microstructural Analysis

**Figure 13:**
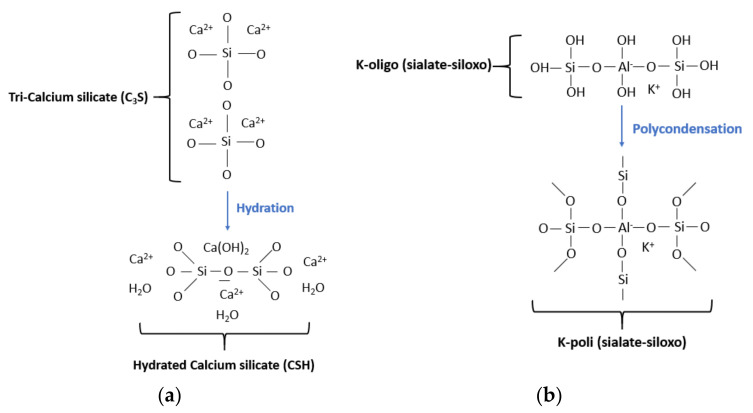
Hardening mechanism in PC (**a**) and geopolymer cement (**b**) [37].

Figure 13 compares the hardening mechanism of PC (Figure 13a) and geopolymer (Figure 13b), in accordance with the models proposed by Davidovits [37]. PC hardens following a hydration reaction of Ca-silicates, the most abundant mineralogical constituents of clinker that predominantly affect the mechanical performance of the material. The hydration results in a CSH phase, which plays a key role on the binding properties of the paste. Conversely, geopolymer cement setting takes place through a polycondensation process of type K-oligo sialate-siloxo into silico-aluminate 3D network, including NASH and K-silico-aluminate hydrate (KASH) phase.

**Figure 14:**
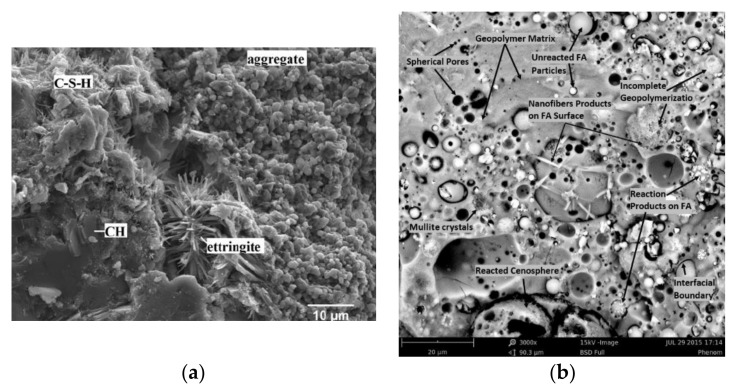
Comparison between (**a**) PC Reprinted with permission from Ref. [70]. Copyright 2013 Microscopy Society of America; and (**b**) FA-based geopolymer cement microstructures. Reprinted with permission from Ref. [71]. Copyright 2016 CRC Press.

The microstructural characteristics of both Portland and geopolymer cementitious matrices are strongly influenced by process parameters, raw materials, and mix design. To appreciate the microstructural differences between the two binders, the microstructures proposed by Guedes et al. [70] and Nuruddin et al. [71] are taken as exhaustive references to compare Portland and FA-based geopolymer cements, respectively. In Portland matrix (Figure 14a), the CSH phase can be identified, the density and homogeneity of which depend on the maturation degree of the paste. Portlandite (CH) and ettringite (CAH) crystals are secondary hydration products. CH fraction results from the hydration reaction of Ca-silicates and represents 20–30% of the hydrated cement paste. CAH fraction results from the reaction between aluminates and Ca and S ions when gypsum is added to the mix. CH phase covers the cement grain, slowing down the hydration rate setting of the cement. Geopolymer matrix (Figure 14b) shows a nano-porous gel that coats large quantities of unreacted or partially reacted (cenospheres) FAs, indicating rapid matrix hardening and incomplete dissolution of precursors. The interfacial bonding between FA and geopolymer phase is predominantly loose, probably resulting from a small amount of aluminosilicate gel developed following alkaline activation. Mullite (3Al_2_O_3_·2SiO_2_) is the major mineralogical phase generated from FA activation.

### 4.2. Geopolymer vs. Ordinary PC Mixes: Mechanical Strength Properties

**Figure 15:**
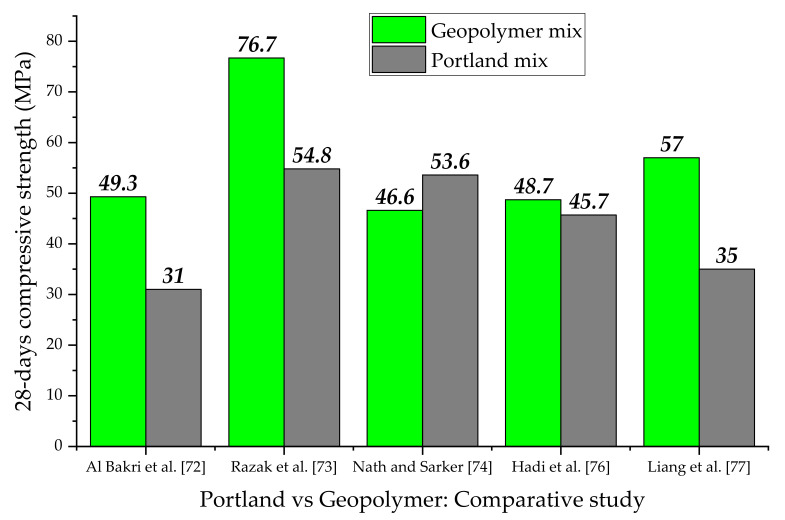
Comparison between Portland and geopolymer mixes in terms of strength properties [72,73,74,76,77].

In terms of compressive strength, the mechanical results of five comparative studies between geopolymer and Portland mixtures are summarized in Figure 15. By optimizing the mix design and the geopolymerization parameters, it is possible to obtain mechanical strengths overall superior to ordinary Portland concrete mixtures. 

Al Bakri et al. [72] compared Portland and FA-based geopolymer concretes, evaluating the influence of various mineral aggregates content in the mixes. The optimal geopolymeric formulation was composed of 30% FA and 70% aggregates, reaching a strength value of 49.3 MPa. For Portland concrete, the higher strength was achieved at 50% of the aggregate amount (31 MPa). However, the mineral aggregates content was not mainly responsible for the strength performance. Indeed, by investigating the geopolymer concrete with 50% of aggregates, a higher strength value (~40 MPa) was still recorded than in the Portland counterpart. The authors attributed the best performance of the geopolymer mixes to the lowest capillary porosity than ordinary cement (3–5% in Portland concrete and 1–2% in geopolymer concrete).

Razak et al. [73] considered the same curing pre-treatment (ambient temperature for 24 h after casting) to evaluate the mechanical performance of FA-based geopolymeric and Portland pastes of fixed composition. The higher mechanical strength found in geopolymer material was also justified in this case by the different microstructural characteristics of the binders under study. FA-based geopolymer paste was denser and more compact than the Portland one, with a pore structure in micropore range (median pore size of 0.8 nm at 28 days). Contrary, Portland matrix mainly showed mesoporosity with a median pore size of 5.3 nm. The authors revealed that the creation of a mesoporous paste provided adverse effects on the strength and the water permeability of the material, reducing its durability. After 28 days, the water absorption was 0.63% and 1.18% for geopolymer and Portland pastes, respectively.

Nath and Sarker [74] researched eleven ambient-cured geopolymer concrete mixtures by considering the water/solid ratio as a study variable, investigating different FA-GBFS replacement levels and the amount of alkaline activator solution (Na_2_SiO_3_-NaOH). For 28 days compressive strength, values ranged from 25.6 MPa (0% of GBFS) up to a maximum value of 46.6 MPa (15% GBFS), indicating a remarkable effect of the Ca-rich additive on the strength development. By incorporating GBFS in the geopolymer mix, improvements in microstructure and mechanical strength are obtained, as the formation of the CSH gel and material densification are promoted [75].

Similarly to previous research, Hadi et al. [76] evaluated the performance of twenty-eight FA-GBFS geopolymer pastes, investigating four factors on the compressive strength: GBFS content (from 10% to 40%), Na_2_SiO_3_/NaOH mass ratio (1.0, 1.5, 2.0, 2.5), and solution-to-binder ratio (0.09, 0.12, 0.15). The optimum mix proportion (GBFS content of 40%, Na_2_SiO_3_/NaOH mass ratio of 2.0, solution-to-binder ratio of 0.15) provided a higher strength value than reference Portland paste, meeting the rheology and setting requirements defined by technical standards.

Liang et al. [77] used rice husk ash (RHA)-MK blends in different proportions to evaluate the mechanical properties of geopolymer compounds with respect to ordinary Portland mixes. All mix designs developed in this study showed greater compressive strengths (ranging from 30.2 MPa to 57 MPa) than the comparison Portland sample. The higher value was found in the mix containing 70% MK coupled with 30% RHA, indicating an optimum balance between filling effect of RHA microparticles for the microcracks and micropores in geopolymer matrix and enriched gel phase formation due to the MK alkali activation. This evidence was demonstrated by BJH pore analysis, whereby increasing the dosage of RHA, a refinement in the pore structure of geopolymer paste occurs. The pore volume in the “best” RHA-MK mix (0.088 g/cm^3^) was smaller than that of the Portland sample (0.100 g/cm^3^), demonstrating the better mechanical performance of geopolymeric formulations. 

### 4.3. Geopolymer vs. Ordinary Portland Mixes: Durability Performance

The service life of concrete structures is intimately related to the material’s durability. According to Tang et al. [78], durability is defined as the ability of cementitious materials to resist weathering action, chemical attack, abrasion, or any other process of deterioration to remain its original form, quality, and serviceability when exposed to its intended service environment. Therefore, the performance of a cementitious mix is not limited to the determination of its mechanical strength, but it is of paramount importance to analyze the material in terms of quality indicators that evaluate its durability, including permeability to various aggressive agents, resistance to acid attack, behavior to freeze-thaw cycling, and others. Table 5 lists the results of some comparative studies between geopolymer and Portland compounds, highlighting the best or worst performances of the geopolymeric mixes compared to ordinary concretes.

#### 4.3.1. Acid Attack Resistance: Discussion

Organic and mineral acid sources involved in the durability of concrete structures include animal secretions, acidic wastewater, acid rain, and silage effluents, which are mainly related to urban, industrial, and agriculture applications [79,88]. From Table 4, geopolymers appear less susceptible to acid attack compared to Portland mixes. This can be attributed to the higher stability of aluminosilicate structure and its relatively low Ca-content. Indeed, Ca-rich phases in Portland matrices (CH, CAH, CSH) are highly vulnerable to the acid environments, forming Ca-based soluble products that leave the cement paste and resulting in porosity and strength degradation [79,80].

#### 4.3.2. Sulphate Attack Resistance: Discussion

Sulphate attack results from the reaction between sulphate ions and hydration products of cement media, which promote the structure damaging by cracking, spalling, softening, and strength loss. Sulphate ions are present in groundwater, soil, and seawater [89]. As reported in Table 4, geopolymer mixes show excellent inertia to sulphate attack with respect to Portland compounds. In the last ones, the exposition to sulphate environment leads to the dissolution of portlandite and decalcification of CSH gel due to the formation of gypsum and CAH crystals, which cause the expansion and cracking in Portland matrix. The low-Ca concentration in geopolymer materials implies a lower aptitude to form the crystal phases mentioned above, resulting in better resistance to deterioration [81,82].

#### 4.3.3. Carbonation Resistance: Discussion

Carbonation of concrete materials involves physical-chemical processes in which a series of chemical reactions occur in the presence of CO_2_, resulting in the reduction of pH in concrete. Altering the material’s alkalinity makes ineffective the protection for the steel reinforcement by initiating the destruction of the passive coating on the reinforcing bars [90]. Table 4 shows worse durability properties towards carbonation for geopolymer concretes than Portland ones. This evidence can be explained by several findings deduced in Portland media: (a) CSH gel has intrinsic resistance to carbonation and the reaction with liquid-phase CO_2_ is very slow; (b) the presence of CAH crystals leads to the formation of insoluble CaCO_3_ phases which reduces the overall porosity of the material [83]. In geopolymer materials, the penetration of CO_2_ produces Na_2_CO_3_ or K_2_CO_3_ components that are highly soluble in water and easily dissolvable, increasing the porosity and permeability characteristics [91]. Pasupathy et al. [91] confirmed the lowest performance of geopolymer concretes than Portland mixes by recording a strong difference in terms of carbonation coefficient. They found a value of about 16 mm/year in FA-based geopolymer concrete in comparison to a value in the range of 1.06–3.54 mm/year for ordinary Portland concrete mix.

#### 4.3.4. Water Sorptivity: Discussion

Water sorptivity index is widely used to evaluate the capillary absorption properties of construction materials. Water is one of the main causes of degradation as it penetrates in the cementitious medium, transports corrosive substances, and freezes inside [92]. According to the data reported in Table 4, the lower sorptivity in geopolymer mixes is consistent with the better performance in terms of acid and sulphate attack resistance than in Portland ones. As also discussed in Section 4.2, geopolymerization appears to develop a denser microstructure than Portland hydration, additionally implying a reduction in the average pore size. These aspects are consequently related to a lower permeability of the cement paste [81,85]. Ganesan et al. [81] found sorptivity index in the range of 21.1–28.5 × 10^−3^ mm/min^1/2^ in geopolymer samples compared to 76.9 × 10^−3^ cm/min^1/2^ recorded in an ordinary Portland mix. Lavanya and Jegan [85] determined a sorptivity index range from 1 mm/min^0.5^ to 3.5 mm/min^0.5^ compared to the range from 1.5 mm/min^0.5^ to 4.5 mm/min^0.5^ found in Portland formulations.

#### 4.3.5. Freeze-Thaw Resistance: Discussion

Freeze-thaw resistance of concrete is of great importance, especially in cold climates, and is one of the main causes of the deterioration of structural materials. The frost resistance is affected by several factors, including porosity, characteristics of the aggregates, and environmental conditions [93]. The worst freeze-thaw resistance in geopolymer mixes can be traced back to the divergence in pore structure and stiffness between CSH gel (Portland mix) and NASH gel (geopolymer mix). Zhao et al. [86] detected different microstructural characteristics between NASH and CSH gels. NASH phase was rich in transition pores (pores between the products of geopolymerization) which negatively affected the material compactness, inducing poorer freeze-thaw inertia than the CSH-based Portland phase. Since the geopolymeric matrices are generally poor in Ca content, the water does not participate in the formation of hydration products such as CSH gel and, by evaporating, it generates highly deleterious pores for the frost resistance. Belforti et al. [87] observed two different mechanical behaviours between geopolymer and Portland mixes subjected to 27 freeze-thaw cycles: a drop in elastic modulus for geopolymer samples and an increase in the Portland one. We hypothesize that the aluminosilicate matrix is intrinsically more sensitive to freezing cracking, reducing the durability of the material.

### 4.4. Geopolymer vs. Ordinary Portland Mixes: Fire Resistance and High-Temperature Behavior

As previously reported (see Table 2), according to specific Si/Al ratios, geopolymer compounds with improved fire resistance and properties suitable for civil applications exposed to high temperatures can be designed. Fire resistance and maintenance of structural stability under hostile thermal conditions are crucial requirements for the serviceability and safety of concrete structures. Thermal conductivity, strength retention, combustibility, and temperature capability are some indicators for the fire/heat endurance of cement materials [94]. The fire behaviour of Ordinary Portland concrete is articulated into two phenomena: thermal and chemical. About thermal alteration, over 400 °C, stress states are generated due to the differential expansion coefficients of the cementitious paste and aggregates. The aggregates tend to dilate while the cement matrix is subjected to thermal shrinkage. This situation induces microcracks in the cement paste, which tend to concentrate near the cement-aggregate interface. Chemically, the thermal evolution of Portland concrete under fire conditions involves the following steps: (a) evaporation of free water contained in the porosity (100 °C); (b) decomposition of CH phase (>350 °C); (c) decomposition of CSH hydrate gel (>500 °C). These transformations lead to an increase in the porosity degree, the concrete strength falling. Due to loss in mechanical properties, at temperature over 600 °C, Portland concretes are considered structurally not suitable [95,96]. To explore the high-temperature peculiarities of geopolymer technology, some comparative investigations with traditional Portland concrete are reported below.

#### 4.4.1. Relationship between Thermal Stability and Pore Structure 

Lahoti et al. [96] reviewed the performance of geopolymeric mixes for structural fire-resistance applications by critically discussing its properties subjected to elevated temperature exposure. Although several factors should be carefully controlled, including choice of precursors, alkali molarity, type of aggregates, and water content, geopolymer materials seem to show superior fire resistance with respect to Portland-based binders. The main findings of the comparative analysis are summarized in Table 6.

#### 4.4.2. Effect of the Alkaline Activator on Fire Performance

**Figure 16:**
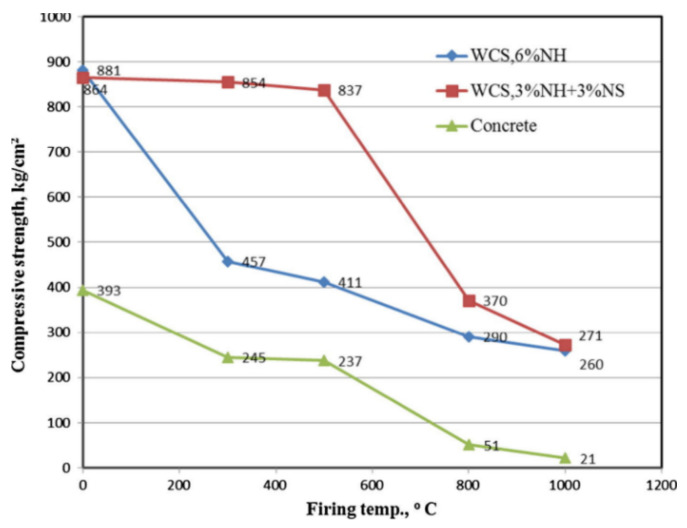
Fire resistance, in terms of strength performance, of MK-based geopolymer and conventional concretes. Reprinted with permission from Ref. [97]. Copyright 2018 Elsevier.

Abdel-Ghani et al. [97] compared the fire performance of MK-based geopolymer binders to Portland mortar by studying different activator solutions: 6% NaOH (labelled in this work as “WCS, 6% NH”) and 3% NaOH + 3% Na_2_SiO_3_ (labelled in this work as “WCS, 3% NH + 3% NS”). The results revealed higher thermal stability and fire strength of geopolymer compounds than conventional concrete, with better properties found in the formulations activated with the binary alkaline activator which confers more ceramic behaviour to the material. At 1000 °C, the strength loss rate was about 95%, 70%, and 68% for ordinary concrete, WCS, 6% NH mix, and WCS, 3% NH + 3% NS, respectively (Figure 16). According to these results, the authors propose the geopolymer mixes as fire-resistant coatings to construction panels for walls, roofs, or partitions. 

#### 4.4.3. High-Temperature Performance of Ferrochrome Slag-Based Geopolymer Concrete

**Figure 17:**
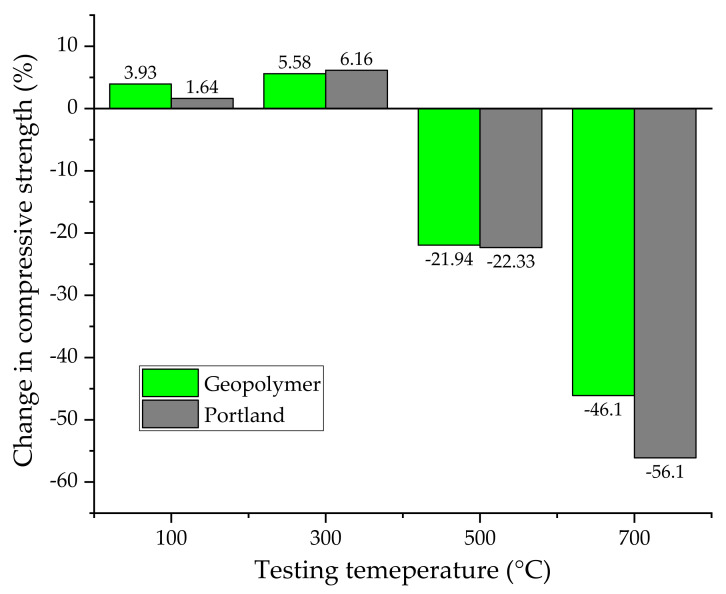
Compressive strength after temperature exposure in Ferrochrome slag based geopolymer and Portland concretes [98].

Turkmen et al. [98] researched the fire behaviour of novel Ferrochrome slag-based geopolymer concrete in comparison with CEM I Portland-based concrete, investigating the strength performance in the temperature range from 0 °C to 700 °C. The most relevant experimental data of this study are reported in Figure 17. Up to 300 °C, an increase in strength was recorded in both samples. For geopolymer, this can be attributed to the thermal-induced promotion of polycondensation in geopolymer gels. For Portland, the temperature increasing tends to accelerate the hydration effect. On the other hand, over 300 °C, both concretes mixes show strength deterioration, which is more pronounced in the Portland formulation. 

#### 4.4.4. Influence of GBFS on Thermal Stability of FA-Based Geopolymer Concrete

**Figure 18:**
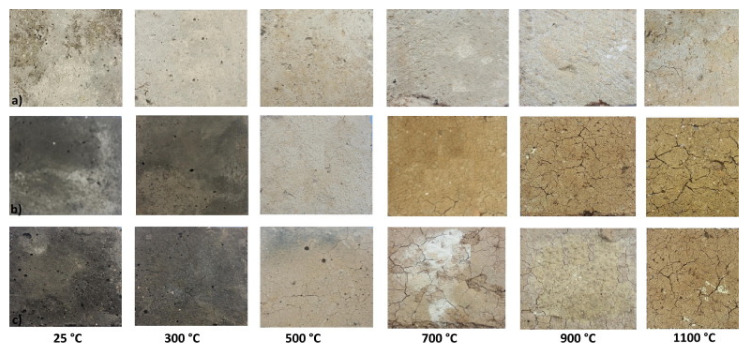
Exposure to elevated temperature of Portland (**a**), FA-GBFS (**b**), and FA (**c**) concretes: texture and cracks distribution. Reprinted with permission from Ref. [99]. Copyright 2017 Elsevier.

Saavedra and de Gutiérrez [99] evaluated the high-temperature performance (300 °C–1100 °C) of alkaline activated mixes based on FA and FA-GBFS blend precursors in comparison with ordinary Portland concrete. For each thermal condition investigated, geopolymeric mixtures showed lower mechanical strength losses than the Portland counterpart. At ambient temperature (25 °C), compressive strengths were 32.0 MPa, 22.5 MPa, and 46 MPa, for FA, FA-GBFS, and Portland mixes, respectively. At 500 °C, the least strength loss (~36%) was found in FA-GBFS mix, while the strongest drop in mechanical resistance (~46%) was recorded in the Portland sample. It is interesting to note that, at 900 °C, Portland concrete suffered a drastic drop in mechanical properties (~85%) and consequent disintegration, making it untestable at 1100 ° C. On the other hand, the geopolymer sample maintained residual strength properties (5.5 MPa and 15 MPa for FA and FA-GBFS mixes, respectively) at the maximum temperature under study. The explanation of this trend agrees with the previous analysis conducted by Lahoti et al. [96]. In Portland materials, a more sudden development of permeable porosity (~11% at 25 °C and ~26% at 900 °C) and consequent mechanical degradation occur compared to geopolymers. Conversely, in geopolymer compounds, high temperatures promoted densification due to the sintering effect of the paste, demonstrating the porosity reduction over 700 °C. The effect of high-temperature exposure on appearance and crack distribution in each investigated sample is reported in Figure 18.

### 4.5. Geopolymer vs. Ordinary Portland Mixes: Energy and Carbon-Emission Analysis

**Figure 19:**
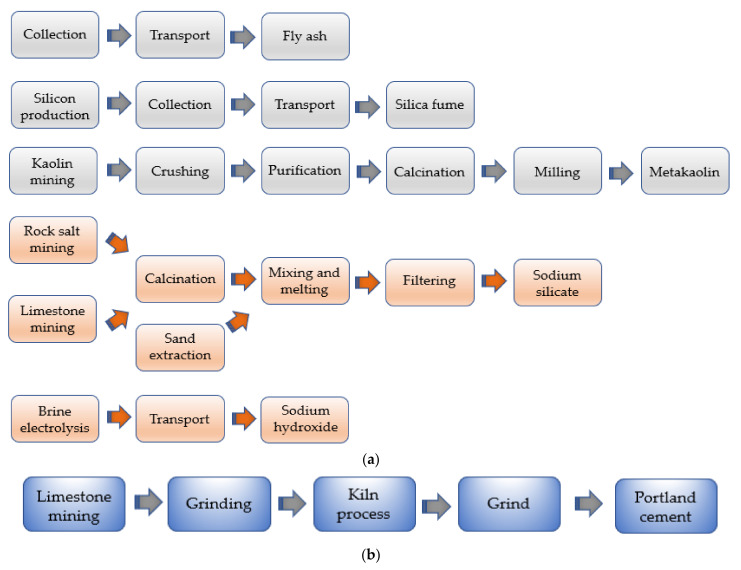
Schematic life cycle stages for production geopolymer (**a**) and Portland (**b**) cements [100].

One of the most attractive aspects of geopolymer technology concerns the beneficial effect on the environment in terms of low carbon footprint and energy consumption, making this class of materials a promising candidate in the concrete industry. In this respect, to assess the impact of geopolymer products, an embodied energy, and CO_2_-emission analysis is reported using ordinary Portland concretes as reference. To better understand the comparative results, the typical life cycle flows of geopolymer and PCs are illustrated in Figure 19, highlighting the crucial phases of energy usage and carbon emissions. Specifically, the models proposed in the McLellan et al. study [100] were considered, which focused on a ternary geopolymeric blend based on FA, MK, and silica fume precursors.

The results of six comparative studies are reported in Table 7. For the data interpretation, some factors that affect the production cycle of the final material must be considered: source location, type of precursors, local availability of raw material, extraction processes, mode of transport, energy source, and number/type of process parameter included in the life cycle assessment (LCA) analysis [106]. 

Although more details about the above LCA studies can be found in the reference papers, some common conclusions can be drawn:

- Although the production cycle of geopolymer materials involves much more articulated stages than the “linear” flow of PC (see Figure 19), overall better performance in terms of CO_2_-emission and energy consumption is recorded, resulting in a valid sustainable alternative to the traditional concrete materials.
- Due to the use of highly alkaline activating solutions, the water ecotoxicity and human toxicity are slightly higher than the PC mix. However, in recent years, more clean approaches were introduced, such as the “one-part” geopolymeric mixes, where the solid aluminosilicate precursors and activators are mixed just with water [107].
- Geopolymer cement production tends to be affected more by the transportation of raw materials than Portland. However, it is important to mention that the influence of transportation depends upon the demographic conditions and local availability of the resource. For instance, the effect in poorly populated countries will be greater than the densely populated countries, assuming that per capita demand and manufacturing of concrete is the same [108].

### 4.6. Section Summary

The purpose of this section was to compare the performance between Portland and geopolymer concrete mixtures based on the mechanical behavior, durability, high-temperature resistance, and environmental impact. By examining past and recent salient comparative studies, interesting benefits of geopolymer-based mixes have emerged. A careful selection of the process parameters allows to obtain superior mechanical properties and greater resistance to aggressive environments. However, concerning the durability properties, the worst carbonation and freeze-thaw cycle resistances were recognized as the main weak points of the material. Further investigation on these aspects will be necessary to optimize the mix design for more durable compounds. Furthermore, due to their ceramic nature, geopolymer mixtures show a marked thermal stability, as opposed to common Portland-based concretes which are chemically unsuitable for exposure to high temperatures. Comparative LCA studies find consensus on the lower carbon footprint and energy impact of the geopolymer production process compared to the clinkering typical of PC concrete mixes. According to the current scenarios of an increasingly “clean” and low-emission cement/concrete industry, this aspect is an added value for the possible implementation of geopolymer technology in the construction sector.

## 5. Conclusions

Among the CO_2_-mitigating technologies intended for the cement and concrete industry, geopolymers have attracted considerable attention both for their sustainability advantages and technological peculiarities. Firstly, this study reviewed the properties of geopolymer mixes, investigating the influence of the synthesis parameters (curing regime, molarity of the activating solution, Si:Al ratio, type of aluminosilicate precursors) on the microstructure, porosity, mechanical strength, and mineralogical characteristics. The “heart” of the manuscript was presented in Section 4, where the authors reported a comprehensive comparative analysis between geopolymer and Portland concrete mixes through more than 40 recent and past literature to explore the potential of using geopolymeric mixes in the field of building materials. From the comparison, valuable information emerged about the applicability of geopolymer-based mixes:

- By optimizing the mix design, comparable/superior strength properties to common Portland concrete mixes can be obtained. The best microstructural quality of the geopolymer matrix, in terms of reduced porosity and strengthening action of aluminosilicate particles, appears to be the main reason for this evidence.
- Overall greater chemical-physical durability (except for freezing and carbonation resistance). The lower permeability and the absence of Ca-rich hydration products (commonly found in Portland pastes) in geopolymer increase the inertia of the material against the permeation of corrosive agents and acid/sulphate attacks.
- Higher thermal stability. The ceramic nature of the geopolymers, instead of the hydrate feature, provides better resistance to high temperatures than PC mixes, allowing application in thermally hostile environments.
- In terms of sustainable production, some comparative LCA data show that geopolymer technology has a lower environmental impact than Portland manufacturing. According to the technical information reviewed in this work, carbon emission and energy consumption are reduced from 10% to 83% and from 50% to 67%, respectively. The variability is due to the availability of raw materials, type of cement formulation, and energy resources used in the production process.

The stability of a geopolymer mix, depending on numerous factors (curing time, molarity of activator, mix design, and precursors composition) is certainly a crucial aspect regarding its diffusion and applicability. Recent research revealed that the incorporation of nanomaterials as functional fillers in geopolymer formulations confers a significant improvement effect on the physical-mechanical properties, especially in terms of mechanical strengthening, microstructural quality, and improved durability. However, the dosage of nano-sized fillers requires special attention to avoid adverse effects on technological performance.

Future research will be directed to the investigations of these process parameters in greater depth to obtain “cleaner” formulations with a good level of reproducibility. Besides, many innovative implementations of geopolymer technology have taken hold in the last few years which will require further investigations: geopolymer foams for thermo-acoustic insulation applications [109], fiber-reinforced composites [110], and alkali-activated pastes for 3D printing [111].

## Figures and Tables

**Table 1:** Effect of different bacterial species on strength performance of cement materials.

Bacterial Specie	28 Days Compressive Strength (MPa)	Strength Rate Increase (%)	Ref.
*Bacillus sphaericus*	6.1–7.3	3.4–23.4	[31]
*Bacillus subtilis*	62.1	20.3	[32]
*Bacillus pasteurii*	42.1	30.3	[33]
*Bacillus cereus*	33.8	11.0	[34]

**Table 2:** Some applications of geopolymeric materials based on Si:Al ratio [47].

Si/Al Atomic Ratio	Applications
1	Bricks—Ceramics
2	Cement—Concrete
3	Heat resistant composites (600–100 °C)—Foundry equipment
>3	Sealant materials (200–600 °C)

**Table 3:** SEM Morphology and EDX analysis of FA-based geopolymer microstructures in relation to alkali concentration and curing temperature. Reprinted with permission from Ref. [50]. Copyright 2016 Elsevier.

Process Parameters		SEM Analysis	EDS Analysis	
*Alkali molarity*	*Curing temperature*	*Morphological features*	*Si/Al ratio*	*Na/Al ratio*
6 M	27 °C	Spherical particles with partially reacted surface. Few Na-rich elongated grains (<5 μm size). Open pores throughout the matrix.	1.3–1.7	0.2–3.0
6 M	45 °C	More reacted surface with reaction product. Numerous Na-rich elongated grains (5–10 μm size). Open pores and few fibrous grains.	1.3–1.7	0.2–4.0
6 M	60 °C	Surface fully altered into reaction product. Numerous Na-rich fibrous grains (up to 20 μm size). Lower pores amount.	1.3–1.6	0.4–5.0
8 M	27 °C	Well reacted surface with reaction products. Al-rich short prismatic grains (2 μm size) occurring in clusters on broken cenosphere. Na-rich dense gel.	1.2–2.1	0.5–3.5
8 M	45 °C	Fully reacted particles enveloped with reaction product. Numerous Na rich fibrous grains (up to 20 μm size). Na-rich dense gel.	1.3–2.1	0.3–3.5
8 M	60 °C	Fully reacted particles, reaction product on the crust. Numerous Na-rich fibrous grains larger than the fibers obtained at 45 °C. Low porosity and compact structure.	1.3–2.1	0.15–3.5
10 M	27 °C	Fully reacted particles covered with reaction product. Al-rich short prismatic grains (2 μm size) occurring in clusters. Na-rich dense gel.	0.6–2.3	0.15–3.5
10 M	45 °C	Fully reacted particles covered with reaction product. Fibrous grains (up to 50 μm size). Na-rich dense gel.	1.1–3.0	0.12–3.8
10 M	60 °C	Fully reacted FA particles. Na-rich gel particles are fusing together to form a dense structure. Si-rich very fine particles (0.5–2 μm size).	1.2–4.0	0.2–3.5

**Table 4:** Influence of alkali molarity on porosity and strength of geopolymer cement, according to several literature data (optimal values in each study are shown in bold).

Ref.	Molarity Level (M)	Porosity (%)	Compressive Strength (MPa)
[57]	6—**8**—10—12—14	21.5—**20.0**—22.5—22.2—22.5	32.5—**36.0**—27.5—27.5—27.5
[58]	12—**16**—18	11.6—**11.1**—11.4	18.0—**27.0**—21.0
[59]	6—8—**10**—12—14	19.0—24.0—**17.5**—18.0—17.8	1.0—9.0—**14.5**—8.0—6.5

**Table 5:** Durability performance of geopolymer mixes compared to Portland concrete.

Durability Indicator	Test Description	Aluminosilicate Precursor	Geopolymer Performance	Ref.
Acid attack resistance	Exposure to 0.1–0.5 M organic acid solution (acetic and lactic acids) for 56 days and evaluation of compressive strength changes and percentage of mass loss	FA	Better	[79]
Acid attack resistance	Exposure to sulfuric acid and hydrochloric acid (pH = 3) for 2 years and evaluation of compressive strength changes and percentage of mass loss	Waste-glass powder (WGP)	Better	[80]
Sulphate attack resistance	Exposure to 3% sodium sulphate solution for 6 months and evaluation of compressive strength changes and percentage of mass loss	FA	Better	[81]
Sulphate attack resistance	Exposure to 10% solution of magnesium sulfate for 1 year and evaluation of compressive strength changes and percentage of mass loss	FA-GBFS	Better	[82]
Carbonation resistance	Accelerated carbonation test: treatment in a carbonation chamber (CO_2_ mass fraction of 20%, temperature of 20 ± 2 °C, and relative humidity of 70 ± 5%) for 2 months and evaluation of carbonation depth	GBFS	Worst	[83]
Carbonation resistance	Accelerated carbonation test: treatment in a carbonation chamber (CO_2_ concentration of 1%, temperature of 23° C, and relative humidity of 65%) for 6 months and evaluation of carbonation depth	FA-GBFS	Worst	[84]
Water sorptivity	Immersion in water for 2 h and gain mass measurement at regular interval of 30 min, resulting the penetration of the water into the material	FA	Better	[81]
Water sorptivity	Immersion in water for 45 days and gain mass measurement at regular interval of 30 min, resulting the penetration of the water into the material	FA	Better	[85]
Freeze-thaw resistance	Exposure to 125 freeze-thaw cycles, according to ASTM C666 method. Hereinafter, evaluation of compressive strength changes and percentage of mass loss was performed	FA-GBFS	Worst	[86]
Freeze-thaw resistance	Exposure to 3 h temperature cycling from −18 °C to 4 °C for 27 cycles. Hereinafter, evaluation of compressive strength changes percentage of mass loss was performed	FA	Worst	[87]

**Table 6:** Main observations of comparative analysis conducted by Lahoti et al. [96] about fire resistance properties of geopolymer and Portland concretes.

Indicator	Portland Concrete	Geopolymer Concrete
Combustibility	Incombustible	Incombustible
Thermal conductivity range	1.0–4.0 W/mK	0.2–0.4 W/mK
Thermal stability	Portland is a hydrate compound. Degradation of hydration products (CH, CSH, CaCO_3_) occurs between 200 °C and 800 °C.	Maintenance of microstructural stability under high temperatures. Geopolymer cement has a glass (ceramic-like) structure and the resistance to heat/fire is better.
Porosity development	Porosity degree gradually increase as a function of increasing thermal stressing, inducing drop in mechanical strength.	Between 500 °C and 900 °C generally occurs the densification of the matrix: geopolymer gel sinters, causing a reduction in void fractions, stronger bonding between particles, and increase in strength.
Pore structure	The chief cause for spalling is the rise in pore pressure due to common close porosity in Portland concrete matrix.	Geopolymer concrete has more interconnected pores: it suggests that water vapor could escape from the geopolymer matrix more quickly than in PC concrete, resulting in lower internal pore pressure and low damage after vaporization.
Degradation condition	Risk of spalling from 200 °C. Structurally not useful above 600 °C.	Lower amount of chemically-bound water implies less susceptibility to spalling. The decomposition mode is to melt over 1000 °C, rather than explosively release water or dehydrate to a powder in Portland concrete.

**Table 7:** Overview of LCA literature data on the carbon footprint and embodied energy involve in geopolymer and PC production.

Geopolymer Cement		PC		Ref.
*CO_2_ emission*	*Embodied energy*	*CO_2_ emission*	*Embodied energy*	
271–404 kg/ton of cement	/	760 kg/ton of cement	/	[100]
239.8 kg/m^3^ of concrete	/	418.8 kg/m^3^ of concrete	/	[101]
*/*	0.33 GJ/ton of concrete	/	1.01 GJ/ ton of concrete	[102]
150–250 kg/ton of cement	2.2–2.4 GJ/ton of cement	800–900 kg/ ton of cement	4.0–4.4 GJ/ton of cement	[103]
320 kg/m^3^ of concrete	/	354 kg/m^3^ of concrete	/	[104]
197.2–210.9 kg/m^3^ of concrete	/	371.7–381.2 kg/m^3^ of concrete	/	[105]
